# Anti-restriction functions of injected phage proteins revealed by peeling back layers of bacterial immunity

**DOI:** 10.1038/s41467-025-63056-3

**Published:** 2025-08-22

**Authors:** Sukrit Silas, Héloïse Carion, Kira S. Makarova, David Sanchez Godinez, Surabhi Haniyur, Lucy Volino, Wearn-Xin Yee, Andrea Fossati, Danielle Swaney, Michael Bocek, Eugene V. Koonin, Joseph Bondy-Denomy

**Affiliations:** 1 Department of Microbiology and Immunology, University of California, San Francisco, San Francisco, CA USA; 2 Gladstone Institute of Virology, J. David Gladstone Institutes, San Francisco, CA USA; 3 National Center for Biotechnology Information, National Library of Medicine, National Institutes of Health, Bethesda, MD USA; 4 Cardiovascular Research Institute, University of California, San Francisco, San Francisco, CA USA; 5 Department of Biochemistry and Biophysics, University of California, San Francisco, San Francisco, CA USA; 6 J. David Gladstone Institutes, San Francisco, California, USA; 7 Quantitative Biosciences Institute, University of California, San Francisco, San Francisco, CA USA; 8 Department of Cellular and Molecular Pharmacology, University of California, San Francisco, San Francisco, CA USA; 9 Twist Biosciences, South San Francisco, CA USA

**Keywords:** Phage biology, Microbial genetics, Bacteria, Bacteriophages

## Abstract

Virus-host competition drives evolution of diverse antivirus defenses, but how they co-operate in wild bacteria and how bacteriophages circumvent host immunity remains poorly understood. Here, using a functional screening platform to systematically explore the functions of phage accessory genes, we describe how cell-surface barriers can obscure the phenotypes of intracellular defenses in *E. coli* isolates. LPS modification emerged as a major theme, with the discovery of several small phage proteins that modify specific O-antigen structures, removing barriers to phage adsorption. Bypassing O-antigen in wild *E. coli* strains revealed another layer of defense: Type IV restriction endonucleases (RE) that target modified DNA of T-even phages (T2, T4, T6). We further show how injected proteins Ip2 and Ip3 of T4 inhibit distinct subtypes of these Type IV REs. Extensive variability in Type IV REs likely drives the emergence of subtype-specific inhibitors through multiple rounds of adaptation and counter-adaptation.

## Abstract (web summary)

Virus-host competition drives evolution of diverse antiviral defences in bacteria and antidefense systems in their viruses (phages). Here, Silas et al. use a functional screen of phage accessory genes to show how bacterial cell-surface sugars can be major determinants of phage host-range, and how some phage proteins injected into bacterial cells inhibit host immunity.

## Introduction

Recent systematic surveys of virus-exclusion mechanisms in prokaryotes revealed a cornucopia of distinct antivirus defense systems^1–5^. Phage-host co-evolution evidently drives diversification of these systems^6–10^. Some defenses strategies (like CRISPR-Cas^11^) exhibit especially diverse repertoires, with a corresponding diversity of virus-encoded anti-defense effectors (such as anti-CRISPR proteins^12^). However, specific phage factors that counter-act many other bacterial defense systems remain elusive, especially, in the context of phage infections in the wild where multiple defenses often operate together^6,13^.

**Fig. 1:**
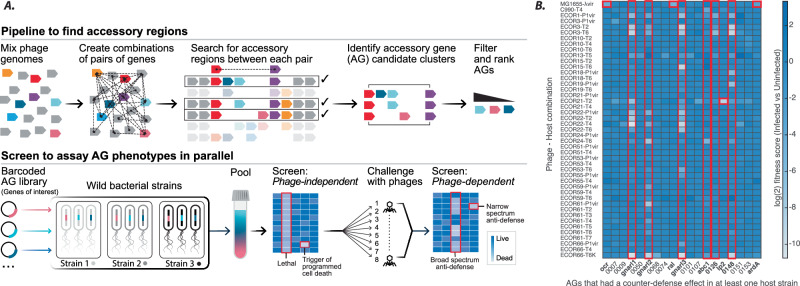
High-throughput screening to find anti-defense phage AGs. **A** Schematic of a computational pipeline to identify phage AGs (described in detail elsewhere^14^), and high-throughput screening platform to assay AG functions in multiple wild strains of bacteria. **B** Heatmap of log2-transformed fitness scores comparing infected and uninfected samples. Of 196 AGs tested, only AGs with a host-sensitizing phenotype are shown on the X-axis. All genes listed on the X-axis were tested in at least one host by plaque assays performed in parallel by two different experimenters, and hits thus validated were further verified by additional plaque assays in triplicate. Red boxes/bold names highlight counter-defense AGs selected for further study. Named AGs are *orf1:ocr, orf48:gnarl1, orf63:gnarl2, orf87:ral, orf92:gnarl3, orf116:abc1, orf143:ip2* and *orf169:ardA*. Mean fitness scores are presented from three independent replicates of the entire screen (see Supplementary Data 1).

We recently built an integrated computational and experimental platform to identify the strategies employed by phages to thwart immunity in wild bacterial strains. Wild isolates more accurately represent the pan-genomic diversity of a species especially with respect to anti-phage immunity mechanisms, which can vary substantially between strains^13^. By systematically testing diverse phage accessory genes (AGs) in diverse *E. coli* isolates, we discovered that the same AG-encoded proteins which inhibit specific immune systems can also trigger programmed-cell-death mechanisms in bacteria^14^. Here, we present the results of parallel screens where we explore how diverse AGs affect phage susceptibility in *E. coli* isolates (Fig. 1A). We report the discovery of phage AGs that encode proteins to compromise a multi-layered host defense architecture, including barriers to phage adsoprtion on the cell surface and bacterial restriction enzymes.

Lipopolysaccharides (LPS) are among the first cellular structures encountered by phages. The outermost LPS structure is a highly variable polysaccharide known as O-antigen. At least 176 distinct O-antigen structures have been identified in *E. coli* and are used to serotype Gram-negative strains for epidemiological purposes^15^. O-antigens play roles in virulence, niche adaptation, and escape from predation^16^. The remarkable diversity of these surface polysaccharides likely evolved through a multifaceted interplay between bacteria, their environment, animal hosts, and their viruses^17^. In the context of phage-host competition, O-antigens can obscure phage receptors on the outer membrane^18^, but some phages, such as T5, can use specific O-antigen structures as primary receptors^19^. Following a successful infection, some phages can also modify O-antigens in a process known as seroconversion^18,20^. The seroconversion strategies identified so far involve enzymes that typically mimic enzymes of the host’s own O-antigen biosynthetic pathway^21–26^.

Should the O-antigen be permissive for cell adsorption and receptor access in a given phage-host pair, DNA injection follows, with some phages also injecting proteins into the host. Although the specific functions of most of these proteins remain enigmatic, they are hypothesized to disrupt host-encoded antiviral defenses. Bacteriophages of the *Tequatrovirus* genus (with prominent members T2, T4, and T6) contain several non-essential genes that encode “internal proteins” (Ip). Phage T4 carries three such proteins, Ip1, Ip2, and Ip3, that are encoded in two distinct loci. Related phages encode many more Ips, with at least 10 reported so far^27^. These internal proteins contain a characteristic N-terminal signal sequence that directs their packaging into the capsid^27^, and they are injected into the host cell along with phage DNA at the onset of infection^28^. Many *ip* genes had been identified in T-even phage genomes by detection of the signal sequence, but only one had been functionally characterized: Ip1 in phage T4 is an inhibitor of a Type IV restriction endonuclease (RE) known as GmrSD, which specifically cleaves glucosyl-hydroxymethylcytosine (g-hmC)-modified DNA typical of T-even phages (T2, T4, T6)^29^.

Here, we report that O-antigen is a critical barrier to phage infection in wild *E. coli* isolates (but not K12-derived lab strains, which lack O-antigen^15^), but that several small phage proteins can alter O-antigen structures without using known seroconverting enzymes. These genes were observed in our AG screens as they serendipitously enhanced the sensitivity to *Tequatrovirus* and other phages (which do not naturally encode them). However, a second line of defense awaits these phages inside the bacterial cell, namely, a diverse repertoire of Type IV REs with GmrS-like effectors. T4-like phages therefore encode a suite of apparently non-homologous inhibitors of these systems (Ip1, Ip2, Ip3) that block distinct GmrSD variants in a non-overlapping way. These outcomes were initially only discerned in wild bacterial isolates once O-antigen barrier defenses were removed or overwhelmed. We further explore the diversity of GmrSD systems that co-evolve with injected proteins of T4-like phages.

## Results

### Phage AGs sensitize bacteria to infection

To discover new mechanisms by which phage AGs increase phage fitness against recalcitrant hosts, we infected 20 wild *E. coli* isolates^30^ with 8 model phages and selected 45 phage-host combinations where phage replication was highly attenuated compared to growth on BW25113 (100–1000 fold, although the attenuation was typically more severe; see Supplementary Fig. 1a). We excluded phage-host combinations where no plaquing or zone of clearing was observed to help ensure at least partial compatability with phage receptors. This phenotype suggests that an antiviral mechanism was blocking phage infection, such that expressing a phage gene that inhibits the relevant defense in the host could alleviate restriction. To this end, we employed a high throughput screening system to bioinformatically identify diverse phage accessory genes (AGs) from highly variable regions of phage genomes, and test their functions in multiple bacterial strains in parallel^14^ (Fig. 1A). We induced the expression of 196 distinct phage AGs in a pooled format, in each strain represented in the 45 phage-host combinations to identify genes that enhanced phage reproduction (Supplementary Data 1). This experiment was executed in triplicate. Although only 8 model phages were used in our infection-based screens, the AGs were selected from an analysis of 1706 Enterobacteriophage genomes as described previously^14^.

To identify AGs with putative counter-defense phenotypes, we compared infected samples with uninfected controls and identified several host-phage-AG combinations that were depleted from the infected pools (Fig. 1B). Each AG that enhanced infection by any phage in a wild strain was then individually conjugated into that strain and tested against all 8 phages by plaque assays. Single plaques were often not observed in many phage-host combinations, making it impossible to calculate precise phage titers and efficiencies-of-plaquing (EOP). Therefore, we measured phage infection scores, defined as the last 10-fold dilution of the phage lysate that still produced a clearing on the bacterial lawn.

Because these validation experiments were performed with the entire phage set, we observed additional phage-enhancing effects of some counter-defense AGs in phage-host combinations that were omitted in the initial screen (Supplementary Fig. 1b). Some AGs, which had produced intermediate fitness defects in liquid-culture screens (e.g., *orfs 7, 68, 107, 151, 153*; see Fig. 1B) failed to reproducibly increase phage titer on relevant ECOR strains in plaque assays. Such discrepancies between liquid and solid culture screening have been observed previously^31^. In all, increased plaquing of several phages was observed in 28 phage-host combinations upon expression of 3 known Type I R-M inhibitors (Ocr, Ral, ArdA)^32,33^, 6 previously uncharacterized AGs found in phages of *Escherichia, Salmonella, and Klebsiella* (Supplementary Data 2), and the T4 internal protein 2 (Ip2)^27^. These AGs likely antagonize host defenses that block phage adsorption or reproduction.

**Fig. 2:**
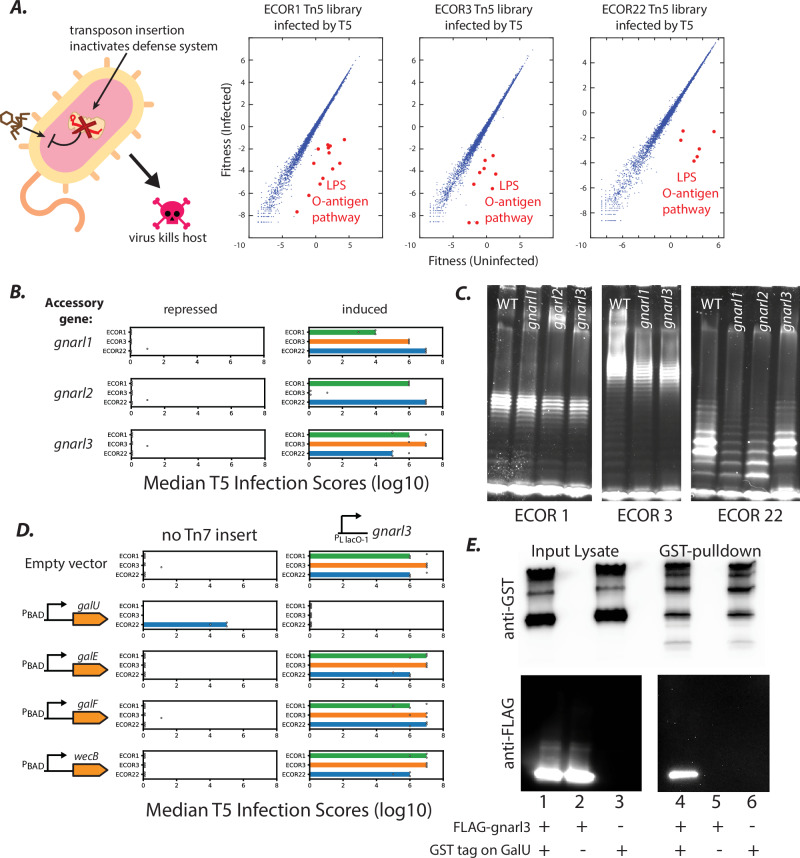
Phage AGs that compromise O-antigen barrier defenses on the cell surface. **A** Schematic of follow-up transposon screens, performed in native hosts without AGs. Graphs show fitness of transposon mutants in infected (Y-axis) and uninfected (X-axis) Tn5-libraries from three representative hosts challenged by T5. Red dots are gene-disruptions that lower bacterial fitness upon infection (e.g., disruptions in O-antigen biosynthesis). **B** Median T5 infection scores in wild hosts ECOR1 (green), ECOR3 (orange), ECOR22 (blue) with *gnarl* genes (*orf48:gnarl1, orf63:gnarl2, orf92:gnarl3*) repressed (left) or induced (right). Infection scores from triplicate plaque assays are depicted by circles. **C** Lipopolysaccharide (LPS) visualized by electrophoresis (*n* = 1) from host-AG combinations in (**B**) where AGs produced a phage-sensitizing effect. (**D**) As in (**B**), Median T5 infection scores with or without *gnarl3* in ECOR hosts, with UDP-glucose biosynthesis pathway genes *galU, galE, galF*, and Enterobacterial Common Antigen (ECA) precursor *wecB* cloned onto a plasmid and overexpressed. **E** GST-immunoprecipitation and western blotting (*n* = 2) with anti-GST (white background) and anti-FLAG (black background) antibodies to test association of Gnarl3 and GalU proteins. GalU (lane 2) or GST-GalU (lanes 1, 3) expressed from a plasmid (pBAD), and FLAG-Gnarl3 (lanes 1, 2) expressed from a single-copy chromosomal insertion (pLlacO-1). Input lysate controls were loaded onto the same gels (lanes 1-3) as the GST-IP eluates (lanes 4-6). Anti-FLAG images are from the same Western Blot, but eluate lanes (4-6) were visualized separately with longer exposure times as FLAG signal in input lysates (1-3) was substantially brighter. Source data for (**C**, **E**) are provided as a Source Data file.

To identify bacterial defenses inhibited by AGs, we employed two unbiased approaches: (1) transposon-mutagenesis screens to pinpoint gene disruptions in hosts that phenocopy AG expression (i.e. increased phage reproduction) (Fig. 2A, Supplementary Data 3) and (2) affinity-purification and mass spectrometry (AP-MS) of AGs to identify binding partners in the wild strains (Supplementary Data 4). We performed these assays for every host found to be sensitized to infection by any AG.

### AGs antagonize core bacterial pathways

We first focused on six previously uncharacterized AGs – *orf48*, *orf63*, *orf92*, *orf116* (previously named *abc1*^34^), *orf126*, and *orf148* that increased fitness of several phages in multiple host strains (Fig. 2B, Supplementary Fig. 2a–c). Because the broad-spectrum effects of these AGs did not correlate with the presence of any specific immune systems within the variable defense repertoires of these wild *E. coli* strains (Supplementary Fig. 1c), we sought to determine whether they targeted core cellular pathways.

Genome-wide transposon-mutagenesis screens indicated that disrupting the host O-antigen or capsule genes phenocopied the phage-sensitization effects of all 6 broad-spectrum counter-defense AGs with almost all tested phages (Supplementary Data 3). The most pronounced phenotypes were that transposon-mediated disruption of O-antigen biosynthesis genes (Fig. 2A) or expression of *orf48*, *orf63*, or *orf92* (Fig. 2B) all enhanced reproduction of phage T5 (which does not natively encode these AGs). We hypothesized that T5 attachment to its secondary receptor FhuA^19^ was blocked by the wild-type O-antigen structures in these *E. coli* strains, whereas heterologous AG expression enabled infection by modifying or removing the O-antigen. We therefore examined LPS preparations from every host strain where any AG exhibited a counter-defense phenotype. Hosts expressing *orf48*, *orf63*, or *orf92* (but not *orf116*, *orf126*, or *orf148*) exhibited subtle downshifts in the O-antigen banding pattern (Fig. 2C, Supplementary Fig. 2d), as observed previously in some seroconverting prophages as a mechanism to exclude superinfecting phage^23^. Specifically, the following host-AG combinations showed visible downshifts in O-antigen bands (Fig. 2C): *orf63* and *orf92* in ECOR1, *orf48* and *orf92* in ECOR3, and *orf48* and *orf63* in ECOR22, which were also the host-AG pairs where T5 infection was especially strongly (10^5-6^-fold) enhanced (Fig. 2B). We reasoned that the AGs might interfere with O-antigen biosynthesis, and tentatively (because we do not yet know their direct targets) named *orf48*, *orf63* and *orf92*, *gnarl1, 2*, and *3*, respectively, after the skin-eating demon in the popular TV series *Buffy the Vampire Slayer*.

All known seroconverting prophages modify O-antigens through the activity of large enzymes such as glycosyltransferases and acetyltransferases^21–26^. *Pseudomonas* phage D3 encodes a small gene *(iap*) that blocks the host O-antigen polymerase, but seroconversion requires two other phage enzymes, an O-acetylase and a ß-polymerase^24^. *Gnarl-*encoding phages in our genomic dataset do not encode any such seroconverting enzymes. Furthermore, Gnarl proteins (40aa – 90aa) are much smaller than typical enzymes and thus are unlikely to exhibit any enzymatic activity themselves. These proteins might affect the activity of bacterial O-antigen-modifying enzymes, constituting a distinct mode of host seroconversion by phages. Intriguingly, *gnarl* genes are found in both lytic and lysogenic phages; *gnarl1* and *gnarl3* are present in lytic phages (Supplementary Data 2), whereas *gnarl2* is present in the classical temperate phage Mu (gene E6/Mup07). Gnarl-mediated LPS modification cannot of course enhance entry of *gnarl-*encoding phages into the host; rather, it can provide an advantage to phages that have already established a lysogenic or persistent virulent state^35^, for instance by excluding superinfection from kin-phages or preventing reattachment to cell debris post-lysis. However, our screen results indicate that *gnarl* expression also serendipitously opens the door to phages that do not encode *gnarls* such as T5.

To identify the cellular targets of Gnarl proteins, we analyzed AP-MS data we had generated for all confirmed counter-defense AGs discovered in our AG screens. Each accessory gene product bound distinct host proteins (Supplementary Fig. 3) but no clear patterns were discernable for any AG (*gnarl1, gnarl2, abc1, orf126, Ip2*, or *orf148*) except *gnarl3*. Gene Ontology pathway analysis identified UDP-glucose biosynthesis factors as significantly enriched in the *gnarl3* set, suggesting that *gnarl3* affects a step in this pathway. Among these, GalU catalyzes the formation of UDP-glucose^36^ that is used in the formation of the LPS outer core^37^; GalF regulates the levels of UDP-glucose^38^; and GalE reversibly converts UDP-glucose to UDP-galactose (also an LPS outer core component)^39^. Overexpression of *galU*, but not *galE*, *galF*, or *wecB* (an unrelated control; WecB is involved in the synthesis of enterobacterial common antigen), completely reversed T5-sensitization by *gnarl3* in ECOR1, ECOR3, and ECOR22 (Fig. 2D), suggesting a specific interaction between *galU* and *gnarl3*. We confirmed that Gnarl3 and GalU proteins associate stably by immunoprecipitation (IP) of GST-tagged GalU and Western blotting to assay co-IP of 3xFLAG-tagged Gnarl3 (Fig. 2E). Infection by an unrelated phage T4 was also enhanced by *gnarl3* in some strains and this phenotype was similarly reversed by *galU* overexpression (Supplementary Fig. 4), indicating that the effect of the *gnarl3-galU* interaction was not limited to phage T5. Overexpressing *galU* without *gnarl3* did not affect phage sensitivity except in ECOR22, where it partially potentiated infection for unknown reasons. Coupled with LPS electrophoresis and AP-MS data, these data suggest that *gnarl3* may inhibit GalU, reducing the availability of UDP-glucose and resulting in a modification of the cell envelope that potentiates T4 or T5 infection. More generally, it is notable that the most prevalent and potent phage sensitization phenotype for multiple, distinct AGs appears to hinge on removing barriers on the cell surface through diverse mechanisms.

### O-antigens and Type IV restriction enzymes provide layered immunity

**Fig. 3:**
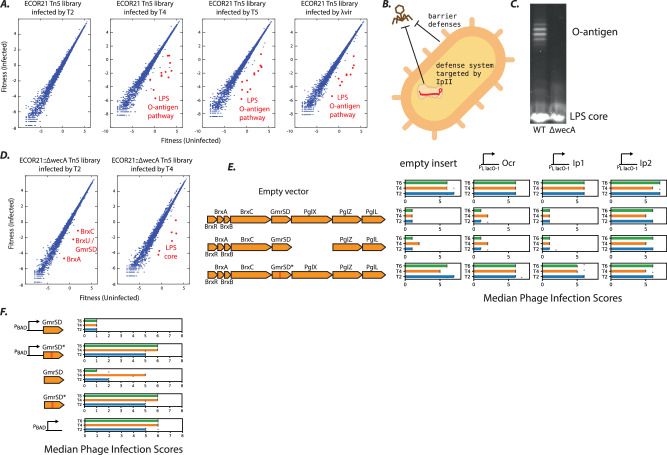
A Type IV RE embedded in a BREX locus. **A** Fitness of transposon-mutants in infected and uninfected Tn5-libraries of ECOR21 challenged by various phages. **B** Schematic of ECOR21 defense against T2 through both barrier defenses and a defense system which is putatively blocked by *orf143*/Ip2. **C** LPS visualized (*n* = 1) from WT ECOR21 (left), and ECOR21 with the O-antigen biosynthesis initiator *wecA* deleted by allelic exchange (right). **D** Whole-genome transposon-mutagenesis screens as in (**A**) but with ECOR21::∆*wecA*. **E** Variants of the BREX/GmrSD operon cloned onto a plasmid along with their native promoters and tested in a lab strain of *E. coli* (TOP10) against T-even phages (T2, T4, T6) in triplicate plaque assays. Removal of the PglX gene inactivates BREX. Active-site mutations that disable GmrSD are shown as two parallel red bars (GmrSD* double mutant; D474A, H475A). Ocr, Ip1 and Ip2 are expressed from single-copy chromosomal insertions. Bar graphs show median log10 infection scores. **F** GmrSD and its inactive variant GmrSD* cloned onto a plasmid and tested in TOP10 for restriction activity against T-even phages as in (**E**). “pBAD” indicates the defense construct was overexpressed from the plasmid (no “pBAD”: same promoter but no arabinose induction). Infection scores from triplicate plaque assays are depicted by circles. Source data for (**C**) are provided as a Source Data file.

Next, we focused on the four other AGs that produced counter-defense phenotypes in our screen. As the targets of Ocr, Ral, and ArdA are known^32,33^, we performed whole-genome transposon-mutagenesis in strain ECOR21, where Ip2 increased fitness of phage T2. However, this was not solely due to an O-antigen barrier, as disruption of the O121-specific O-antigen pathway^40^ sensitized the strain to T5, Lambda vir, and T4, but not T2 (Fig. 3A). We wondered if this discrepancy could be explained by the existence of an additional defense mechanism in ECOR21 which complements the O-antigen barrier by restricting T2 infection (Fig. 3B). In the AG screen, internal-protein Ip2 partially sensitized ECOR21 to T2 (Fig. 1B), suggesting that T2 is blocked by an Ip2-inhibited system, in addition to O121 O-antigen. Although phage T2 itself does not encode Ip2, related phage T4 encodes Ip2 as well as the only internal-protein from T-even phages with a known function, Ip1. Ip1 is packaged into the phage capsid and disables a Type IV RE upon co-injection with phage DNA^29^ (the original system from *E. coli* strain CT596 was previously misannotated; it consists of a single GmrSD protein, and not two separate proteins GmrS and GmrD^41^).

To identify the Ip2-inhibited system, we eliminated ECOR21 O-antigen by deleting *wecA*^42^ gene (Fig. 3C, Supplementary Fig. 5) and repeated the whole-genome knockout screen, looking for transposon mutants in the ECOR21∆*wecA* background that became sensitized to T2. With T2 infection, several sensitizing transposon insertions emerged in an atypical BREX system^43^ (Fig. 3D, Supplementary Data 3) in which a GmrSD enzyme (also known as BrxU) is embedded^44^. Notably, the same screen with T4 did not yield these hits, suggesting that this system is not an impediment to the Ip2-encoding T4. The entire ECOR21 BREX-GmrSD system cloned into a lab strain and expressed from its native promoter blocked T2 infection, but an active-site mutant in GmrSD (denoted GmrSD*) did not (Fig. 3E). By contrast, inactivation of the BREX system (∆*pglX*) had no impact on T2 inhibition, confirming that GmrSD alone inhibits T2 and is the likely target of Ip2. Consistent with this conclusion, expression of Ip2, but neither Ocr (which inhibits BREX and Type I R-M^45^) nor Ip1 (which inhibits GmrSD in *E. coli* CT596^29^), abolished GmrSD/BrxU defense (Fig. 3E).

To test for sufficiency, we expressed GmrSD from ECOR21 or its catalytically inactive variant GmrSD* alone in the lab strain. Overexpressing GmrSD was sufficient to block all T-even phages, whereas the catalytically inactive mutant had no effect (Fig. 3F). Leaky expression of GmrSD from the uninduced plasmid still inhibited replication of T2 and T6 (which do not encode Ip2), but not the Ip2-encoding phage T4 (Fig. 3F). Of the many described T-even phage internal proteins^27^, Ip2 is only the second (after Ip1) with an identified target.

### A distinct Type IV RE requires two GmrS-like genes

We wondered whether the observed redundancy between cell-surface barriers and restriction systems obscured phenotypes of other anti-restriction AGs as well. Although the wild strain ECOR17 was not included in our AG screen, Srikant et al. recently observed that mutants of phage T4 in which Ip3 was eliminated lost their ability to infect this strain and hypothesized that a defense system in ECOR17 targeted T4 but was blocked by Ip3^46^. We performed genome-wide transposon screens to identify this system, reasoning that its disruption would render ECOR17 susceptible to infection by Ip3-deficient T4.

**Fig. 4:**
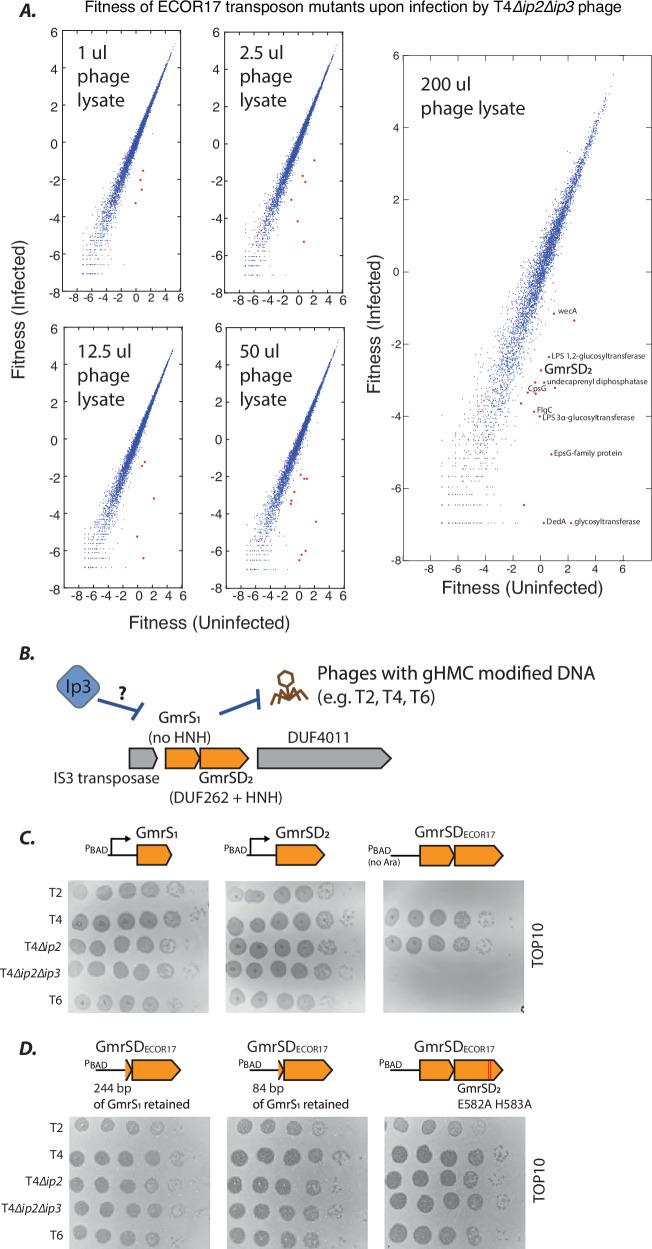
A Type IV RE with a GmrS/GmrSD doublet. **A** Fitness of the same transposon-mutant library of ECOR17 (2.31e7 cfu) challenged by various amounts of a ~ 5.5e9 pfu/ml lysate of phage T4∆*ip2*∆*ip3*. Tn5 insertions that compromise phage resistance (red dots) in low-phage-input screens are listed in Supplementary Data 3. **B** Schematic of ECOR17 locus that encodes defense system putatively blocked by Ip3. **C** Plaque assays to measure anti-phage activity of GmrS_1_ and GmrSD_2_ components individually (with overexpression) and of the full system (without overexpression). T4 and T4∆*ip2* phages naturally encode Ip3. **D** Plaque assays to measure anti-phage activity of constructs (without overexpression) with truncated GmrS_1_ or GmrSD_2_ with E582A H583A mutations in its active site. Images in (**C**), (**D**) are representative images from plaque assays performed in triplicate.

Various transposon insertions in ECOR17 facilitated infection by a T4 mutant lacking both *ip2* and *ip3* genes (Supplementary Data 3), including disruptions of genes responsible for biogenesis of cell-surface polysaccharides, particularly O-antigens. We hypothesized that O-antigens were again obscuring an underlying defense system. Since we struggled to obtain ∆*wecA* mutants of several ECOR strains for unknown reasons, we repeated the transposon screens with increasing concentrations of phage to attempt to overwhelm and bypass the surface barrier (Fig. 4A). In general, higher phage concentrations resulted in more significant hits while still recapitulating the hits found at lower concentrations. We found that transposon insertions in a GmrSD gene (containing a signature DUF262 domain^41,44^ homologous to the N-terminal NTPase domain (ParB_n) of ParB-like proteins involved in a variety of defense mechanisms, and an HNH nuclease domain) were sensitized to infection by T4∆*ip2*∆*ip3*, but only at high phage concentrations (Fig. 4A, Supplementary Data 3). Inspection of this genetic locus revealed an unusual Type IV RE with two GmrS homologs, GmrS_1_ and GmrSD_2_ (Fig. 4B). Both genes encode a ParB_n domain, but only GmrSD_2_ (identified in our transposon screen) contains an HNH endonuclease domain (Supplementary Fig. 6).

We sought to determine whether both GmrS genes were required for phage defense. We cloned the genes in an expression vector into a lab strain of *E. coli* and tested their ability to provide defense against model T-even phages (T2, T4, and T6) and T4 mutants lacking either *ip2*, or both *ip2* and *ip3*. Overexpression of either GmrS_1_ or GmrSD_2_ alone provided no protection against any of the phages, and inducing both genes together (hereafter, GmrSD_ECOR17_) resulted in gross cellular toxicity. However, the GmrSD_ECOR17_ construct was tolerated well without induction, and provided robust defense against T2, T6, and T4∆*ip2*∆*ip3*, while T4 and T4∆*ip2* could both still replicate (Fig. 4C). This result suggested that leaky expression of the two-gene GmrSD_ECOR17_ construct was sufficient to restrict replication of T-even phages that do not encode *ip3*.

We considered the possibility that GmrSD_2_ could provide phage defense without GmrS_1_ but required an upstream regulatory element contained within GmrS_1_ such as one of the two overlapping antisense small ORFs extending up to 84 bp and 244 bp upstream of the GmrSD_2_ start codon respectively. Therefore, we tested GmrS_1_ truncations with only 84 bp or 244 bp upstream of GmrSD_2_ retained in the two-gene construct, but found that neither variant could restrict phage infection (Fig. 4D). We also tested whether the conserved HNH-endonuclease active site in GmrSD_2_ was required, as with other GmrSD nucleases. We found that the endonuclease-dead variant (E582A H583A double mutant) did not provide defense (Fig. 4D). Our findings suggest that two GmrS paralogs in ECOR17 function in concert, perhaps as a protein complex, to restrict T-even phage DNA, likely using the HNH endonuclease domain of GmrSD_2_. GmrSD only digests g-hmC DNA^29^ but BrxU (GmrSD_ECOR21_) can also digest hypo-modified T4 DNA^44^. We tested the substrate preferences of our GmrSD variants and confirmed that only GmrSD_ECOR21_ could restrict hypo-modified T4 phages^47^ (Supplementary Fig. 7).

Overall, Ip2 and Ip3 block GmrSD variants from wild *E. coli* strains (ECOR21 and ECOR17, respectively) that also produce O-antigens which can interfere with phage adsorption and obscure the phenotype of the underlying anti-phage defense systems.

### Injected proteins from T4-like phages block specific subtypes of the vastly diverse GmrSD family

**Fig. 5:**
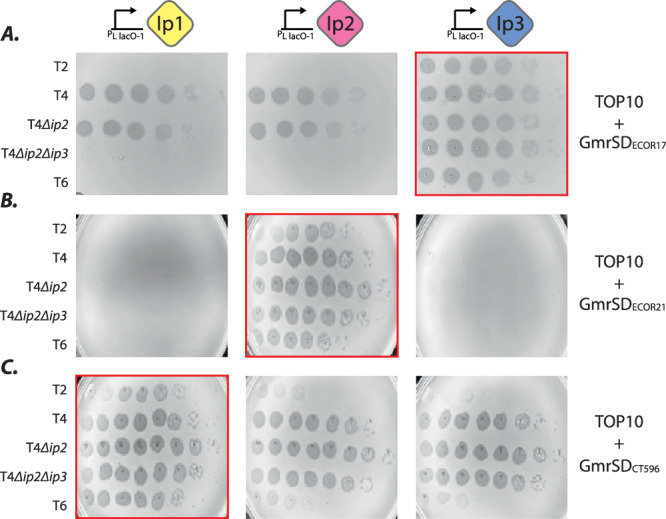
Injected proteins (*ip*) from T4 phage block specific GmrSD variants. Plaque assays to measure anti-phage activity of (**A**) GmrSD_ECOR17_ (CLS_003/004) (**B**) GmrSD_ECOR21_ (CLS_010), and (**C**) GmrSD_CT596_ (CLS_006) with heterologous expression of Ip1-3 (red borders indicate GmrSD variant is paired with its *ip* inhibitor). *ip* genes are expressed from single-copy chromosomal insertions. Ip1 is naturally present in T4, T4∆*ip2*, and T4∆*ip2*∆*ip3*, Ip2 in T4, and Ip3 in T4 and T4∆*ip2*. All plasmid-encoded GmrSD genes were tested without over-expression.

GmrSD_ECOR17_ could not restrict T-even phages that carried Ip3, namely, T4 and T4∆*ip2*. To test whether Ip3 was sufficient to block restriction, we expressed each T4 internal protein (Ip1, Ip2, Ip3) individually in *E. coli* carrying GmrSD_ECOR17_. Ip3 restored plaquing by T2, T4∆*ip2*∆*ip3*, and T6 to wild-type levels, whereas Ip1 and Ip2 had no effect (Fig. 5a). T4 and T4∆*ip2* could infect as usual in all three conditions. Therefore, Ip3 specifically inhibits GmrSD_ECOR17_.

Ip1 and Ip2 of phage T4 are also inhibitors of Type IV REs comprised of GmrSD nucleases, hereafter GmrSD_CT596_^29^ and GmrSD_ECOR21_ (this study)_,_ respectively. We wondered why a single phage would need to carry three distinct GmrSD inhibitors. Given that only Ip3 could inhibit GmrSD_ECOR17_, we tested if any of the T4 internal proteins provided cross-protection. Assaying all three T4 Ips against the targets of Ip1 and Ip2 (GmrSD_CT596_ and GmrSD_ECOR21_), we found that each *ip* gene specifically disabled only one system, with no cross-protection observed (Fig. 5B, C). Therefore, Ip1, 2, and 3 are all Type IV RE inhibitors, but each specifically blocks a distinct GmrSD effector. We note that WT phage T4 was unable to disarm GmrSD_ECOR21_ unless additional Ip2 was supplied exogenously (Fig. 5B), but could naturally overwhelm GmrSD_CT596_ and GmrSD_ECOR17_ in our assays (Fig. 5A, C). This is likely due to a difference in GmrSD expression levels. Since GmrSD_ECOR21_ is embedded in a BREX operon and expressed from its native promoter from a multi-copy plasmid, it is likely present at much higher concentrations in the cell than GmrSD_CT596_ and GmrSD_ECOR17_, which are both extremely toxic and could only be tested under leaky expression conditions (without their native promoters).

To further investigate the specificity of Ip effectors for their GmrSDs, we constructed and examined pairwise alignments of the GmrSD protein sequences. The GmrSD targets of Ip1 and Ip2 are highly diverged (Supplementary Data 5). Similarly, the two components of GmrSD_ECOR17_ are also extremely diverged from GmrSD_CT596_ and GmrSD_ECOR21_ (Supplementary Data 5). Except for the DGQQR motif that is conserved in almost all GmrSD proteins^41^, there was virtually no similarity between any of the sequences.

Previous phylogenetic analysis identified many highly diverged sub-types of GmrSD-encoding Type IV REs^41^. To explore this diversity, we retrieved sequences matching DUF262/ParB_n profiles (pfam03235 and COG1472) from the conserved domain database (CDD) assignments precalculated for 24,757 completely sequenced genomes^48^. To identify major groups within the GmrSD family, the retrieved proteins were clustered by sequence similarity using MMseqs2^49^, and domain composition and gene neighborhoods of the 20 most common GmrSD family proteins (named CLS_001-020) in Enterobacteria were examined in detail to identify potential additional components of these systems (Supplementary Data 6). CLS001-020 GmrSD variants were arranged in two predominant Type IV RE architectures: (a) 12 single-gene systems encoding a GmrSD protein containing a ParB_n domain and an endonuclease domain (from either HNH or predicted nuclease DUF1524 of His-Me finger endonuclease superfamily), and (b) 3 two-gene systems consisting of two *gmrSD* genes from different clusters where one of the components is a two-domain protein containing ParB_n and an endonuclease domain whereas the second gene contains a ParB_n domain only. Of the 12 single-gene systems, two are closely similar to GmrSD_CT596_ (CLS_006) and GmrSD_ECOR21_ (CLS_010), and among the three two-gene systems, one is similar to GmrSD_ECOR17_ (CLS_003/CLS_004).

We synthesized representative systems from all 15 GmrSD groups, and tested them against our panel of T-even phages. Five systems (in addition to GmrSD_CT596_, GmrSD_ECOR21_, and GmrSD_ECOR17_) showed apparent anti-phage activity against T-even phages. We tested ten *ip* genes from T4-like phages against these new systems but identified no new inhibitory interactions (Supplementary Figs. 8,9). Together, the intimate association between *ip* genes and the highly diversified GmrSD enzyme family reveals a strong arms race is underway. Further, these data underscore the importance of O-antigen barriers that can obscure the immune repertoire of wild strains.

## Discussion

Identification of phage AGs and characterization of their interactions with host defenses can tease apart the layers of bacterial anti-phage immunity, providing a holistic view of the salient defense mechanisms across multiple bacterial strains. In a previous work, we showed that phage accessory genes (AGs) can activate bacterial immunity pathways that lead to cell death or dormancy^14^. Here, we extend our high-throughput functional screening platform to investigate how AGs facilitate phage infection by interacting with the multi-layered antivirus defense architecture in wild bacterial strains. Assaying 196 Enterobacteriophage AGs in 20 wild isolates of *E. coli*, we found that AG-encoded proteins modify the cell surface to alter phage sensitivity, disable antiviral restriction systems inside the cell.

The most common counter-defense phenotype in our screens involved “broad-spectrum” AGs that interfered with cell-surface barriers such as O-antigens which are broadly conserved across many strains of the host species (as opposed to strain-specific defense systems). The commonly used K12 lab strain of *E. coli* does not produce O-antigen, but most wild strains of *E. coli* do^15^. By performing our screens in wild *E. coli* isolates, we discovered several AGs (*gnarl1-3*) that modify O-antigens. These phage proteins appear to function via a previously uncharacterized mechanism of seroconversion wherein Gnarls can modify O-antigens in various wild bacterial strains as stand-alone effectors, without the involvement of any known phage-encoded seroconverting enzymes. Indeed, none of the lytic or temperate phages that carry *gnarl* genes encode any such enzymes. Therefore, if LPS modification is the primary function of *gnarl* genes, seroconversion of bacterial hosts by their phages could be a much more widely distributed phage strategy than previously thought^21–26^. O-antigen modification cannot facilitate the entry of the initial *gnarl-*encoding phage into the cell, however, it could benefit lysogens by excluding superinfecting phages, and lytic phages by receptor-masking to prevent newly synthesized virions from unproductively binding fragments of lysed cells (indeed, a similar function is attributed to the Llp protease in phage T5^50^). Persistent infection by lytic phages may also be more common than is presently believed^35^, and seroconversion could be a useful superinfection-exclusion strategy in such instances. Whereas the precise role of *gnarl* genes in the phage lifecycle remains a subject for future work, our AG screening approach allows us to identify such capabilities in the genomes of phages that we do not currently have in the laboratory, and pinpoints which phages should be procured to further study these novel functions.

One of the *gnarl* genes encodes a small phage protein Gnarl3 which binds GalU (UDP-glucose pyrophosphorylase) and its expression results in O-antigen alteration. Although we cannot rule out that Gnarl3 binds to GalU to effect another metabolic function, previous work has identified phages that could not adsorb on *E. coli* with *galU* mutations^51^, nor on *B. subtilis* with mutations in the *galU* homolog *gtaB*^52^, suggesting a widespread, ancient link between glycosylation of cell-surface structures and phage tropism. We hypothesize that O-antigen diversification is driven in part by selection pressures from phage-encoded LPS modification mechanisms^53^ such as interference with GalU function by Gnarl3. More extensive functional genomics screens can be expected to reveal that many phage AGs can cause cell-surface modifications via diverse mechanisms.

By removing or overwhelming the O-antigen barriers in specific wild strains of *E. coli* pinpointed by our AG screen, we discovered that injected proteins in T4-like phages^27^ disable various GmrSD-encoding Type IV REs. In particular, the internal proteins Ip1, Ip2 and Ip3 of phage T4 are all injected into the host cell along with phage DNA and specifically inhibit different GmrSD systems. The enormous diversity of *gmrSD* genes and the strict specificity of their respective inhibitors could explain why a single phage (T4) encodes multiple inhibitors (Ip1-3) of the same family of defense systems. Type IV restriction is evidently a major frontier in the arms race between Enterobacteria and *Tequatrovirus*.

Our discovery of the inhibition of specific Type IV RE systems by Ip1-3 prompted us to test whether these genes might also block other GmrSD variants. We found that Ip1-3 specifically inhibit only their cognate GmrSD enzymes. Apparently, extensive co-evolution of Type IV REs with T-even phages has driven the emergence of new GmrSD variants, as well as the evolution of the corresponding diversity of injected anti-defense proteins in this phage genomic hotspot. Ip1-3 share no sequence homology other than an N-terminal leader sequence that directs their packaging into capsids. Notably, the GmrSD nucleases they target also share extremely limited sequence homology, suggesting that these inhibitors might make extensive contacts with their target enzymes, as was observed in structural studies with Type I R-M inhibitors^33^. As in the case of anti-CRISPR proteins co-resident in genomic hotspots in a *Pseudomonas* phage family^54^, it seems likely that the accessory proteins encoded in the two *Ip* loci in T-even phages all target different variants of the same bacterial immune system, namely, GmrSD-encoding Type IV restriction enzymes.

In summary, we show that O-antigens in wild host strains obscure the phenotypes of counter-defense phage AGs at subsequent stages of infection. The GmrSD targets of Ip2 and Ip3 in phage T4 had remained elusive for decades^27^ and overcoming O-antigen barriers in strains that naturally encode these GmrSD enzymes was necessary to observe underlying anti-phage immunity. Incompatibility between diverse O-antigens and phage adsorption mechanisms is not considered a canonical mechanism of phage defense. However, its dominant role in systematic screens with wild bacterial strains suggests that this incompatibility may well turn out to be one of the most important variables governing the success of phage therapy. That several phage AGs can affect O-antigen modification in wild strains in a manner that serendipitously benefits phages we used in our screens further suggests that a limited set of seroconversion strategies could be employed strategically to potentiate adsorption of a variety of different phages in a therapeutic cocktail. This leaves the door open for a reductionist and rules-based approach to phage therapy design.

## Methods

This section contains expanded versions of previously published protocols; where appropriate, we have provided the protocols in entirety for completeness, retaining relevant text from the original^14^. Details of key materials are provided in Supplementary Table 1.

### Strains and vectors

The *E. coli* Reference (ECOR) collection of wild *E. coli* strains was obtained from the STEC Center (Michigan State University). Lab strains were obtained as follows: MG1655 (from Dr. Carol Gross, UCSF), C990 (from Dr. Ry Young, Texas A&M), DH5α (NEB), DH10ß/TOP10 (Life Technologies), BW25113 and BW25141 (from Dr. Vivek Mutalik, LBNL), and WM6026 (from Dr. Jason Peters, UW-Madison). All strains were cultured at 37 °C with shaking (180–225 rpm) in lysogeny broth (LB) (10 g/l tryptone, 5 g/l yeast extract, 10 g/l NaCl with 15 g/l agar for plates) supplemented with 100 µg/ml of ampicillin/carbenicillin, 10–20 µg/ml gentamicin (except WM6026, which yielded more consistent colony sizes with 50 µg/ml), 50 µg/ml kanamycin, and 0.5% w/v glucose, 0.1% w/v L-arabinose, and 1 mM IPTG (isopropyl-b-D-thiogalactopyranoside) as needed. The WM6026 strain required diaminopimelic acid (DAP) for growth, which was supplied at 300 µM. All strains were stored in 20% glycerol at −80 °C for long term storage.

R6K-origin pTn7 plasmids (from Dr. Jason Peters, UW-Madison) – the pJMP1039/pTn7C1 helper plasmid (carbenicillin) and the pJMP1360/pTn7C185 transposon vector (gentamicin, carbenicillin) – were used for integration of phage AGs into the *E. coli* genome. IPTG was used in all cases as the inducer for phage AGs from the plLacO-1 promoter encoded on pJMP1360/pTn7C185 and integrated into the host genome by Tn7 transposition (see below). All host defense systems identified in the various screens were placed under the araBAD promoter (arabinose inducible) in the pBAD Myc/His A vector (Invitrogen). Conjugative allelic exchange was carried out using R6K-origin pKEK2201 (kanamycin) (from Karl E. Klose, UT San Antonio). All plasmids were constructed by Gibson Assembly.

R6K-origin plasmids were maintained in BW25141 (routine cloning) and WM6026 (conjugative donor strain). All other plasmids were maintained in DH5α or TOP10. All plasmid sequences were verified by Sanger or whole-plasmid sequencing (Plasmidsaurus, Primordium) and are available upon request. Frequent plasmid dimerization during the Gibson Assembly procedure (especially when using the NEBuilder reagent) was ameliorated by using 10-fold less of one of the insert DNA fragments in a reaction involving at least 2 insert fragments (not including the backbone).

### AG conjugation into host strains

AGs were delivered to *E. coli* hosts by Tn7 transposition via conjugation performed by triparental mating as described previously^55^. Briefly, the transposon and helper plasmids were delivered to the recipient from WM6026 donor strains by overnight incubation on LB agar plates supplemented with 300 µM DAP and 0.5% glucose to repress AG expression. The next day, cells were scraped and resuspended in 1 ml LB, and 1000X-20000X dilutions of this conjugation mixture were plated on LB plates (lacking DAP) supplemented with the appropriate antibiotics and 0.5% glucose. All strains were maintained under strict glucose repression of the pLlacO-1 promoter at every step until screens were performed. Thus, all AGs were maintained as single-copy chromosomal inserts at the same attTn7 site in the genomic region between *glmS* and *pstS* genes.

Efficient Tn7 transposition is necessary to construct an AG library in any given *E. coli* host. Tn7 conjugation-transposition efficiencies were determined in 44 ECOR stains containing an intact *glmS-pstS* region (we note that other strains can support Tn7 integration as well). Tn7 transposons carried only a barcode sequence and the gentamicin-resistance gene to minimize payload-dependent variation in conjugation efficiency between strains. Efficiency of conjugation was determined by streaking the conjugation mixture on two different gentamicin concentrations (25 µg/ml and 10 µg/ml) to account for possible differences in natural antibiotic resistance between the wild strains. The barcode sequence was verified by Sanger sequencing and both flanks were checked by PCR to ensure proper integration of the Tn7 transposon at the expected attTn7 site. Strains that supported Tn7 transposition at high levels and were amenable to agar-overlay plaque assays were selected for further experiments.

### DNA extraction and nextera whole-genome sequencing

Genomic DNA was extracted using a modified SDS–proteinase K method: Briefly, cells pelleted from 50 µl of saturated culture (or 5 µl of the cell mixture scraped from selective plates during AG library construction) were re-suspended in 200 µl of lysis buffer (10 mM tris, 10 mM EDTA, 400 µg/ml proteinase K, 0.5% SDS) and incubated at 50−55 °C for 1 hour. Subsequently, the temperature was lowered to 37 °C and RNase A (Thermo Scientific) was added to a concentration of 1 mg/ml. After 30−60 min of incubation, the digests subsequently purified using the Genomic DNA Clean & Concentrator Kit (Zymo Research). DNA was prepared for whole-genome sequencing using the Nextera Flex Library Prep Kit (now, the Illumina DNA Prep Kit) according to manufacturer’s instructions, except that we lowered the volumes of all tagmentation reactions 5-fold, and used a custom dual-indexed primer set JSW-SS-22:33 (CAAGCAGAAGACGGCATACGAGAT NNNNNNNN GTCTCGTGGGCTCGG) and JSW-SS-34:41 (AATGATACGGCGACCACCGAGATCTACAC NNNNNNNN TCGTCGGCAGCGTC) to amplify libraries for 11-13 cycles using Phusion High Fidelity PCR Master Mix (NEB) instead of the Illumina-supplied PCR reagents. The N8 sequences correspond to reverse-complemented Nextera DNA indexes N701 to N712 and N501 to N508, respectively (Illumina). Libraries were resolved by agarose gel electrophoresis and DNA was excised in the 300-400 bp range. Gel slices were purified using the Zymo Gel DNA Recovery Kit and sequenced on the Illumina MiSeq using 150-cycle v3 or 600-cycle v2 reagent kits.

Sequencing adaptors were trimmed from reads using cutadapt, mapped to reference genomes using bowtie2, and visualized using IGV. Reference genomic contigs of ECOR strains were obtained from the USDA^56^.

### Phage propagation and plaque assays

High-titer phage stocks were generated by growth in liquid culture. All phage infections were performed at 37 °C in LB supplemented with 10 mM MgSO_4_ and 5 mM CaCl_2_. *E. coli* BW25113 or C990 were grown to an optical density (at 600 nm; OD_600_) ~ 0.5 (1−5 × 108 cfu/ml) and infected with the desired phage at MOI ~ 5. Infections were allowed to proceed at 37 °C with agitation for 5−6 h until the cultures were clear. Lysates were clarified by centrifugation (8000 × g for 2 min) and sterile-filtered using a 0.45 µm SFCA syringe filter (Millipore). Phage lysates were stored at 4 °C.

Bacteriophages T2, T3, T4, T5, T6, T7, P1vir were obtained from Dr. Vivek Mutalik (LBNL), and λvir was obtained from Dr. Ry Young (Texas A&M). To the extent possible, all phages were grown on *E. coli* C990 which lacks restriction-modification. T4 and P1vir could not be grown on C990 and were instead grown on *E. coli* BW25113. To ensure that our phage lysates were free from ancestral Type I R-M methylation marks or other host-passage derived DNA modifications, we serially passaged all phages in the desired hosts 3 times. All phage genomes were subsequently verified by Nextera high-throughput sequencing.

Phage titers were determined against various hosts by the agar-overlay method with LB top agar containing 0.7% agar, 10 mM MgSO_4_ and 5 mM CaCl_2_. Phage spot-titration assays were performed with 10-fold serial dilutions of phage lysates (3−5 µl of each dilution pipetted onto bacteria immobilized in top-agar overlay using a multichannel pipette).

### AG Library Preparation and NGS verification for testing post-conjugation evenness

200 AGs with unique 10 bp DNA barcodes were synthesized (Twist and IDT) and cloned individually under pLlacO-1 control (IPTG inducible, repressed by glucose) into a modified pTn7C185 vector (with mobile CRISPRi components sgRNA and dCas9 removed). Plasmid assembly reactions were performed in 96-well format using the 2X Gibson assembly master mix (NEB) and transformed into chemically-competent BW25141 cells in 96-well format. Transformations were individually plated on LB agar supplemented with 20 µg/ml gentamicin and 0.5% glucose and one colony was initially checked by Sanger sequencing from each plate. Where a successful transformant could not be obtained on the first attempt, more colonies were sequenced in successive rounds until a plasmid with no mutations in the region spanning the pLlacO-1 promoter, the AG ORF, and the barcode sequence was obtained. Verified vectors were miniprepped using the ZR Plasmid miniprep – Classic kit (Zymo) and transformed into chemically-competent WM6026 cells in 96-well format, and each transformation was plated individually on LB agar supplemented with 50 µg/ml gentamicin, 300 µM DAP, and 0.5% glucose.

One colony from each of the 200 WM6026 transformants was grown in 2 ml square-well 96-well plates with shaking overnight, and 100 µL of each culture was then pooled together to yield ~20 ml of the donor mixture. This mixture was concentrated down to 10 ml by centrifugation at 6000 g for 10 min, and 5 ml of 60% glycerol (20% final) was added to yield the donor library. The 15 ml library was divided into single-use 50 µL aliquots (each containing >50 M cells, i.e., >300 K of each donor strain) and stored at −80 °C.

Genomic DNA was extracted from one of the library aliquots and all constructs in the final pool were verified by Nextera high-throughput sequencing. This allowed us to assess library evenness as well as verify the fidelity of our cloning, colony picking, and strain pooling process. Overall, 195 AGs were cloned successfully. One AG (*orf88*) never yielded any colonies in multiple cloning attempts, possibly due to its cytotoxicity. Three AGs (*orf36, orf52, orf94*) were dropouts during the WM6026 growth step, and another two (*orf25, orf84*) were discovered to contain mutations upon high-throughput sequencing that had been missed by Sanger sequencing. *Orf25* had previously presented difficulties in cloning, and did not yield a mutation-free construct even after checking >12 colonies (whereas most transformations yielded a correct construct on the first attempt). The *orf84* construct contained a mixed base at the last position of the second repeat of the Lac-operator sequence upstream of the ORF, which would not be expected to affect any downstream assays significantly.

The donor library was conjugated into MG1655 *E. coli* according to the Tn7 transposition procedure (above) and a 1000X dilution of the conjugation mixture was plated on LB agar supplemented with 20 µg/ml gentamicin and 0.5% glucose. We routinely use multiple 150 mm × 15 mm bacteriological petri dishes (Corning) to obtain sufficient numbers of transconjugant colonies to assemble libraries. The next day, >>2000–20,000 transconjugant colonies (10-100X the number of AGs in the library) were scraped and combined thoroughly, and the thick cell mixture was directly frozen at −80 °C in 20% glycerol. Genomic DNA was extracted from this mixture and the distribution of DNA barcodes corresponding to individual AGs was measured in both this post-conjugation strain mixture as well as the genomic DNA extracted from the WM6026 donor library aliquot.

To amplify barcodes for high-throughput sequencing, genomic DNA was subjected to two successive rounds of PCR. The DNA barcodes were amplified from genomic DNA using universal primers JSW-SS-170 (CGACGCTCTTCCGATCTNNNNN TGATGTCGTTGTTGCCATCG) and JSW-SS-171 (ACTGACGCTAGTGCATCA CTTTCTGAGCCAGTGTTGCT) and the Q5 Hot Start High-Fidelity 2X PCR master mix (NEB) according to manufacturer’s instructions. Sequencing adaptors were attached in a second round of PCR using amplicons from the first PCR round as the templates, with dual-indexed primer sets JSW-SS-42:53 (CAAGCAGAAGACGGCATACGAGAT NNNNNNNN GTGACTGGAGTTCAGACGTGTGCTCTTCCGATCT ACTGACGCTAGTGCATCA) and JSW-SS-54:61 (AATGATACGGCGACCACCGAGATCTACAC NNNNNNNN ACACTCTTTCCCTACACGACGCTCTTCCGATCT), where the N_8_ sequences correspond to reverse-complemented TruSeq HT indexes D701 to D712 and D501 to D508, respectively (Illumina). Template-matching regions in the primers are underlined. Cycling conditions for round 1 were as follows: one cycle at 98 °C for 1 min; two cycles at 98 °C for 10 s, 66 °C for 30 s, and 72 °C for 10 s; 22 cycles at 98 °C for 10 s, and 72 °C for 20 s; and one cycle at 72 °C for 2 min. Conditions for round 2 were one cycle at 98 °C for 1 min; two cycles at 98 °C for 10 s, 64 °C for 30 s, and 72 °C for 10 s; 4 cycles at 98 °C for 10 s, and 72 °C for 20 s; and one cycle at 72 °C for 2 min. 0.5–1 µL of unpurified 1^st^ round reaction product was used as template for the 2^nd^ round of PCR. Amplicons from the 2^nd^ round were gel-purified by electrophoresis (3% agarose gel, 4.2 V/cm, 2 hours) and quantified using the 1X dsDNA HS kit with the Qubit 4.0 Fluorometer (Invitrogen). Amplicons were sequenced on a MiSeq using the 150-cycle v3 reagent kit (Illumina) in single-end format (100 bp) with two (8 bp) index reads. DNA barcodes were trimmed from the reads and the proportions of various AGs in the mixtures were determined using the prevalence of the corresponding barcode sequence.

Concordance between the Nextera whole-genome sequencing and the barcode sequencing of the WM6026 donor mixture showed that barcode representation was a suitable proxy for the true distribution of AGs in the mixture and that amplification biases during library preparation were not a significant source of noise. Concordance between barcode sequencing in the WM6026 donor and MG1655 post-conjugation recipient mixtures indicated that the Tn7 transposition and colony scraping process does not meaningfully bottleneck AG distribution.

The AG library was then conjugated (in triplicate) into ECOR strains that were previously confirmed to support robust Tn7 transposition. Strain mixtures were prepared in a procedure analogous to library construction in MG1655, except higher dilutions of the ECOR + AG conjugation mixtures (10,000–20,000X) were plated and the gentamicin concentration on selective plates was lowered to 10 µg/ml to account for higher steady state cell densities and higher gentamicin sensitivity of the wild *E. coli* strains. As with the MG1655 library, thick cell mixtures comprised of >>2000–20,000 colonies scraped from each replicate conjugation were directly stored at −80 °C in 20% glycerol and used to seed cultures for the AG screens (below). Genomic DNA was extracted from each mixture and barcode sequencing was performed to measure the AG distribution in every replicate of all ECOR strain derived libraries. The replicates were very highly correlated, and AG distributions in all libraries were confirmed to be free of significant bottlenecking. These datasets are the reference libraries that serve as the baseline for AG induction and phage infection screens.

### MOI calculation for liquid killing assays

Phage adsorption to host cells may be inefficient in very dilute cultures (10^6-7^ cfu/ml). Therefore, the apparent multiplicity of infection (MOI-a) might be lower than the expected MOI (MOI-e). We added 2x serial dilutions of a λvir lysate to a 1:100 dilution of a saturated culture of *E. coli* BW25113 that had been allowed to acclimate at 37 °C with shaking for 15 min. The proportion of surviving cells was determined (relative to uninfected controls) after a 60–75 min incubation (one phage replication cycle) by plating on LB agar. An apparent MOI value was calculated using the Poisson distribution function with zero occurrences ${MOIa}=-{{\mathrm{ln}}}({MOIe})$ for each phage dilution, assuming that all infected cells would be killed by the virus (i.e., the proportion of surviving cells would be the zero class in the Poisson distribution). The apparent and expected MOI values were plotted against each other and the inverse of the slope (1/m) of the line (MOI-a = m*MOI-e + 0) fit through the data was determined as the fold-change reduction in MOI-a as compared to MOI-e for these experimental conditions. This fold-change reduction was determined to be ~26x. Hence, all phage infection screens were targeted to an MOI-a value of ~100 to ensure efficient phage adsorption and infection within the timeframe of a single phage replication cycle.

### AG Screen, data analysis, and verification by plaque assays

Thick cell mixtures from each of the three replicates of the various ECOR + AG libraries were thawed on ice and diluted into LB supplemented with Mg^2+^, Ca^2+^, and 10 µg/ml gentamicin. Typically, 1–5 µl of the library mixture was diluted in 2 ml broth to yield an initial OD_600_ of 0.1–0.15. The cultures were immediately split x2, with one tube used to monitor OD_600_ values as the experiments progressed, and the other left untouched for the screen. AG expression was induced by the addition of 1 mM IPTG after acclimatization for 15 min at 37 °C in a shaking incubator and OD_600_ values were measured again for each ECOR + AG library at time of induction. Libraries were then grown for ~80–120 min depending on the relative growth rates of the various ECOR strains, to OD_600_ ~ 1.2–1.5. A 10^−^^5^ dilution of each late-log phase culture was then plated on LB agar (with 10 µg/ml gentamicin and 0.5% glucose to repress AGs and avoid fitness costs) to obtain a count of viable colony forming units (cfu). Cultures were immediately diluted ~1:20 in fresh LB (with IPTG, Mg^2+^, Ca^2+^, and 10 µg/ml gentamicin) and 1 ml aliquots were gently mixed with phage lysates at an MOI-e of ~30–100 (the actual MOI-e values were recorded the next day when cfu counts were obtained and are indicated in Supplementary Data 1). Parallel uninfected controls were maintained for each experiment, even when the same ECOR + AG library was being tested against different phages and the uninfected controls would be interchangeable. AG induction was limited to 1–2 h and subsequent infections were only allowed to proceed for ~1 phage replication cycle (30–35 min for T-phages and 60–70 min for P1vir and λvir) to prevent prolonged induction of various AGs from biasing the results. For example, this setup also avoids biases resulting from epigenetic modification of phages by widespread restriction-modification (R-M) defenses in *E. coli*. However, we note that allowing sufficient time for the phages to complete cell lysis was necessary as fast-growing cells were sometimes able to form colonies on LB agar despite having been infected. These colonies had an uneven size distribution and a “splotchy” appearance, perhaps due to ongoing infection on the plate that was nevertheless not able to outpace colony formation. After the infection period was complete, the cultures were vortexed, diluted 1:100, vortexed again, and 50 µl of the dilution was plated on 2 large (150 mm × 15 mm) LB agar plates (with 10 µg/ml gentamicin and 0.5% glucose to repress AGs and avoid fitness costs). The next day >>2000–20,000 colonies were scraped from each infected and uninfected set of plates. 5 µl of the thick resulting cell mixtures was reserved for genomic DNA extraction, and the rest was frozen at −80 °C in 20% glycerol as a backup. A full technical record of our screens is provided in Supplementary Data 1.

AG barcodes were amplified and sequenced as with the WM6026 donor library, with a slight modification. The three biological replicate sets of ECOR + AG experiments were amplified using JSW-SS-171 and different versions of primer JSW-SS-170 containing extra N’s at the start of the sequencing read. The first replicate was amplified with JSW-SS-170 (containing 5 N’s), the second replicate with JBD-SS-254 (CGACGCTCTTCCGATCTNNNNNN TGATGTCGTTGTTGCCATCG) with 6 N’s, and the third replicate with JBD-SS-255 (CGACGCTCTTCCGATCTNNNNNNN TGATGTCGTTGTTGCCATCG) with 7 N’s. This ensured that reads from the three sets would not cross contaminate analyses of replicate experiments. Amplicons were sequenced to the same depth as the baseline libraries on Illumina MiSeq and NextSeq instruments in single-end format (100 bp) with two (8 bp) index reads.

We required exact matches for the 20 bp primer-derived invariant sequences flanking the barcodes. These sequences were trimmed and the proportions of various AG-encoding strains in the mixtures were determined using the prevalence of the corresponding 10 bp barcode sequences. Barcode counts per AG were normalized by dividing each count by the median of each experiment, which would represent neutral AG fitness. A log2 transformation was subsequently applied to obtain fitness scores. Care was taken to sequence each library to similar depth such that fitness scores across experiments were distributed in the same range. Poorly represented AGs were identified in the baseline libraries as well as the infected and uninfected experimental samples using a low read cutoff of 100, or if the log2 normalized average read counts across the three replicates for any AG were >3x the median absolute deviation below the median for that experiment. Reproducibility was determined using T-tests across the three replicates, and we enforced a *P*-value cutoff of 0.2. The magnitude of the AG fitness effect was determined by (a) subtracting the AG fitness scores in the uninfected library from the infected libraries to assess whether AGs changed host sensitivity to phage infection, and (b) subtracting the AG fitness scores in the baseline library from the uninfected libraries to assess whether AGs change host fitness directly. AGs were selected for further study if they were not poorly represented to begin with, produced reproducible effects across replicates, and changed host fitness by at least 4-fold. Although we had targeted our experiments to phage-host combinations where the host was mostly resistant to the phage in order to find counter-defense AGs, a few hosts turned out to be more sensitive to certain phages in liquid infection than suggested by agar-overlay plaque assays. In these cases, our platform was also able to robustly identify AGs that conferred a protective advantage to the host (e.g., Imm and Cor, which both block phage DNA entry^57,58^). In practice however, these effects can be very variable and the reproducibility filters typically needed to be relaxed in order to find such “superinfection exclusion” AGs.

All counter-defense AGs were subsequently conjugated into the wild ECOR hosts where they demonstrated an effect, and tested against all 8 phages (not just the ones that were used in the screen with that host). In several hosts, the AG was found to additionally sensitize the host to phages that were omitted in the screen. Not all the screen phenotypes could be confirmed in agar-overlay plaque assays, possibly due to variations in cell growth between liquid and solid media, or due to the requirement for successive complete infection cycles and sufficient burst sizes for the formation of visible plaques that would render plaque assays less sensitive than single-infection cycle dynamics in our screen. Nevertheless, further experiments were performed only in phage-host-AG combinations that could be confirmed by plaque assay due to ease of follow up testing.

Conditional lethal AGs were identified directly from the heatmap of fitness scores, with a subset selected for further experimentation using an arbitrary fitness cutoff of ~6–7 ( ~ 100-fold).

### RB-TnSeq Tn5 library construction and data analysis

Tn5 whole-genome knockout strain libraries were constructed using the RB-TnSeq donor library (pKMW7-derived strain APA766 from Dr. Adam Deutschbauer) conjugated with the recipient ECOR strain in a biparental mating on LB plates supplements with 300 µM DAP. The next day, cell mixtures were scraped and resuspended in 1 ml LB and 1000X dilutions of this conjugation mixture were plated on 10 large (150 mm × 15 mm) LB plates (lacking DAP) supplemented with 50 µg/ml kanamycin. Approximately 0.5–1 million colonies were scraped taking care to break colonies apart on the surface of the plates, mixed very thoroughly, aliquoted and stored at −80 °C with 20% glycerol, with ~5–10 µl reserved for genomic DNA extraction and reference library preparation.

RB-TnSeq allows the same mutant library to be challenged under various conditions such as challenge by various phages, and simplifies the downstream sequencing-library preparation by substituting barcode sequencing for all experimental conditions after initial characterization of the reference library. For completeness, we describe the RB-TnSeq procedures here as implemented in our lab.

To characterize the Tn5-knockout reference libraries in the various ECOR strains, genomic DNA was extracted from 5 to 10 µl of the cell-scrape and prepared for sequencing using the NEBNext Ultra II FS DNA Library Prep Kit (NEB) with the following modifications. 500 ng DNA was fragmented for 25 min according to manufacturer’s instructions. The adaptor ligation step was performed as directed albeit with a custom pre-annealed oligo adaptor comprising of YL001 (/5Phos/GATCGGAAGAG/3ddC/) annealed to one of YL002:005 (AGCGGCAATTTCACACAGGACAAGCAGAAGACGGCATACGAGATNNNNNNNNAGTGGTGACTGGAGTTCAGACGTGTGCTCTTCCGATC*T) where the 4-base underlined sequence serves as a variable barcode unique to each oligo. The USER Enzyme steps post-ligation were skipped and the bead-cleanup was performed using 40 µl and 20 µl of Sample Purification Beads successively as instructed for library size selection in the 150–250 bp range. The PCR amplification of transposon-genome junctions was performed using the cycling parameters described in the kit for 11 cycles with Q5 Ultra II FS Master Mix (NEB) using primers YL006 (AGCGGCAATTTCACACAGGA) and JBD-SS-272/oAD493 (ACACTGGCAGAGCATTACGCCCT) instead of the kit-supplied primers. A second nested-PCR enrichment step was performed for 11 cycles using the same cycling parameters as before, using the entire bead-purified reaction product from the first step as a template with primers YL009 (CAAGCAGAAGACGGCATACGAG) and JBD-SS-273:280 (AATGATACGGCGACCACCGAGATCTACAC TATAGCCT ACACTCTTTCCCTACACGACGCTCTTCCGATCT NNNNNNN GCAGGGATGTCCACGAGGTCTCT) where N_8_ barcodes correspond to reverse-complemented Illumina TruSeq HT D501-508 indexes. Bead-purified amplicons were quantified using the 1X dsDNA HS kit with the Qubit 4.0 Fluorometer (Invitrogen) and sequenced on Illumina MiSeq and NovaSeq instruments in single-end format (150 bp) with two (8 bp) index reads.

The first 100 bp of these reads contain transposon derived sequences and a DNA barcode that uniquely identifies a particular Tn5 insertion. The last 50 bp of these reads contain host-genome derived sequence that help determine the location of the transposon insertion and hence construct a map between the Tn5 barcode and the insertion site (IS) in the genome. Briefly, 20 bp barcodes and 16-50 bp IS sequences were trimmed from the sequencing reads. To isolate the barcode and IS, we allowed 3 mismatches in the 23 bp invariant sequence upstream and up to 6 mismatches in the 50 bp downstream of the barcode using a Hamming distance function to compare the expected and actual sequences. Any reads failing these sequence-fidelity requirements were discarded. Reads containing an IS that matched the Tn5 transposon vector in the donor strain were also discarded. The 4 Ns adjacent to the underlined index sequences in YL002:005 were also sequenced as part of one 8 bp index read and served as a tag to remove PCR duplicates. This allowed us to distinguish between sequencing “reads” and DNA “fragments” present in the sample at the adaptor ligation step. Thus, reads containing unique combinations of barcode, PCR tag, and IS were entered into the barcode/IS map for each Tn5 library, each representing a distinct DNA fragment (but also machine errors in sequencing, chimeric PCR amplicons, 3’ truncations etc.). The filtered ISs were then mapped to genes within their respective host genomes. As described previously^59^, it is possible for transposon barcodes to map to the genomes ambiguously. Barcodes associated with multiple distinct ISs were retained only if their most frequently represented IS accounted for over 90% of the fragments containing that barcode. We then counted the number of DNA fragments recovered per gene and used this as a reference library for subsequent BarSeq experiments to assay depletion of strains with mutations in specific genes upon phage infection.

Tn5 mutant strain libraries of the various ECOR strains were then challenged by various phages in a procedure analogous to the ECOR + AG screens. Cultures of Tn5 libraries were initiated with 100 µl of thick cell scrape mixture in 100 ml LB supplemented with 50 µg/ml kanamycin, Mg^2+^, and Ca^2+^, to an initial OD_600_ value of 0.1–0.15. Cultures were grown at 37 °C with shaking for 80–90 min to an OD_600_ of 0.7–1. A 10^−^^5^ dilution of each late-log phase culture was then plated on LB agar (with 50 µg/ml kanamycin) to obtain a count of viable colony forming units (cfu). Cultures were immediately diluted ~1:10 in fresh LB (with Mg^2+^, Ca^2+^, and 50 µg/ml kanamycin) and 1 ml aliquots were gently mixed with lysates of the appropriate phages at an MOI-e of ~30–100 (the actual MOI-e values were recorded the next day when cfu counts were obtained and are indicated in Supplementary Data 3). Since the parental ECOR strains were typically quite resistant to phage infection to begin with, only mutants with transposon-inactivated defense systems would be expected to drop out upon infection. In practice however, some strains exhibited more pronounced mortality upon prolonged phage infection in the Tn5 screens. In particular, in experiments with T2 and the Tn5 library constructed with the ∆*wecA* strain of ECOR21 (which was generally more susceptible to infection by T2 and other phages), MOI was titrated in three parallel infection screens (with varying amounts of phage lysate added: 350 µl, 100 µl, and 25 µl) to empirically ensure specific depletion of only the phage-sensitive mutants. Similarly, in experiments with T4∆*ip2*∆*ip3* and the Tn5 library constructed with ECOR17 (which resists infection by T4∆*ip2*∆*ip3* using both O-antigen and GmrSD_ECOR17_), MOI was titrated in five parallel infection screens (with varying amounts of phage lysate added: 200 µl, 50 µl, 12.5 µl, 2.5 µl, 1 µl). Uninfected controls were maintained for each experiment, and each set of experiments was performed in biological duplicates. Infections were allowed to proceed for 5 min at the benchtop without shaking, and 75 min at 37 °C with shaking. Since an interaction between the host and phage had already been identified in the earlier AG screen, infections were not restriction to a single phage replication cycle, but allowed to proceed further to achieve full depletion of the phage-sensitive Tn5-knockout mutants in the pool. After the infection period was complete, the cultures were vortexed, diluted 1:10, vortexed again, and 100 µl of the dilution was plated on 2 large (150 mm × 15 mm) LB agar plates (with 50 µg/ml kanamycin). We note that this dilution yields bacterial lawns and in our experience this preserves diversity of surviving colonies without degrading the signal of dropouts by phage-killing in the experiment. The next day, bacterial lawns were scraped from each infected and uninfected set of plates. 5 µl of the thick resulting cell mixtures was reserved for genomic DNA extraction, and the rest was frozen at −80 °C in 20% glycerol as a backup. A full technical record of our screens is provided in Supplementary Data 3.

To amplify barcodes from the post-selection infected and uninfected Tn5 libraries, we followed a modified BarSeq98 method^59^, with two successive rounds of PCR. The transposon barcode sequence was amplified by PCR using Q5 Ultra II FS Master Mix (NEB) with primers JBD-SS-310 (CGACGCTCTTCCGATCTNNNNNNN GATGTCCACGAGGTCTCT) and JBD-SS-311 (ACTGACGCTAGTGCATCA GTCGACCTGCAGCGTACG). Template-matching regions in the primers are underlined. Sequencing adaptors were attached in a second round of PCR using amplicons from the first PCR round as the templates, with dual-indexed primer sets JSW-SS-42:53 and JSW-SS-54:61 (sequences already provided in section describing AG screening). Cycling conditions for round 1 were as follows: one cycle at 98 °C for 4 min; two cycles at 98 °C for 30 s, 63 °C for 30 s, and 72 °C for 30 s; 22 cycles at 98 °C for 30 s, and 72 °C for 30 s; and one cycle at 72 °C for 4 min. Conditions for round 2 were one cycle at 98 °C for 1 min; two cycles at 98 °C for 10 s, 64 °C for 30 s, and 72 °C for 10 s; 4 cycles at 98 °C for 10 s, and 72 °C for 20 s; and one cycle at 72 °C for 2 min. 0.5–1 ul of unpurified 1^st^ round reaction product was used as template for the 2^nd^ round of PCR. Amplicons from the 2^nd^ round were gel-purified by electrophoresis (3% agarose gel, 4.2 V/cm, 2 hours) and quantified using the 1X dsDNA HS kit with the Qubit 4.0 Fluorometer (Invitrogen). Amplicons were sequenced on Illumina MiSeq and NovaSeq platforms in single-end format (150 bp) with two (8 bp) index reads.

DNA barcodes were trimmed from the reads and the proportions of various Tn5-knockout mutants in the mixtures were determined using the prevalence of the corresponding barcode sequence. Briefly, we allowed 3 mismatches in the 18 bp invariant sequence upstream, and 4 mismatches in the 18 bp downstream of the transposon barcodes using a Hamming distance function to compare the expected and actual sequences. The frequency of occurrence of each barcode (representation number) was counted. Transposon barcodes were matched to those found in the reference libraries, inferred to represent knockouts in the genes containing the corresponding ISs, and the various barcode representation numbers were added together for each gene. Cumulative transposon insertions per gene were then compared statistically between the infected and uninfected samples (with the reference library as a baseline to filter out poorly represented, presumably essential genes) using the same data analysis procedure as for the AG screen, albeit with two biological replicates instead of three.

### LPS gel electrophoresis

Lipopolysaccharide was prepared from liquid overnight cultures of wild *E. coli* strains expressing the relevant AGs (LB + IPTG). Briefly, 50–100 µl of saturated culture was spun down and cells were thoroughly resuspended in 100–200 µl of lysing buffer (62.5 mM Tris-HCl, pH6.8, 10% glycerol, 2% SDS, 4% ß-mercaptoethanol). We routinely supplement 1X Laemmli Buffer (BioRad) with SDS and ßME to make lysing buffer. Samples were boiled at 100 °C for 15 min, and allowed to rest at room temperature for 15 min. 100 µg of Protease K were added to each sample and protein was digested at 60 °C for at least 1 h. 15 µl of each sample were run onto a 12% polyacrylamide TGX Mini-Protean Precast gel (BioRad) at 200 V for 35 min, and visualized using the Pro-Q Emerald 300 LPS stain kit (Thermo Fisher) precisely according to manufacturer’s instructions.

### Affinity purification of AGs from wild *E. coli* hosts

FLAG-tagged AGs were conjugated into ECOR strains by Tn7-transposition (above) according to Supplementary Data 4. Each strain was grown overnight at 37 °C in 2 mL of LB supplemented with 0.5% glucose and 10 µg/mL gentamicin, then diluted 1:100 into 100 mL LB supplemented with 10 µg/mL gentamicin and grown at 37 °C for 30 min. The media was then supplemented with 1 mM IPTG and grown for another 2.5 h. “Popcorn” pellets were collected by centrifugation for 30 min at 4000 × g, resuspension in 100 µL of ice-cooled lysis buffer [50 mM Tris pH 7.4, 150 mM NaCl, 1 mM EDTA, 1 mM MgCl_2_, 0.5% NP40 (Research Products International), 125 U/mL Benzonase (Millipore), 1x protease inhibitor cocktail (Roche, cOmplete ULTRA tablets, mini, EDTA-free), 0.5 mg/ml lysozyme (Fisher Scientific)], followed by dropwise addition into liquid nitrogen and stored at −80 °C. Popcorn pellets^60^ were lysed by 10 cycles of cryomilling for 2 min at 12 counts per second in a SPEX SamplePrep 6870D Freezer/Mill and stored at −80 °C.

For each phage-host combination, 3 replicate lysates were reconstituted using 200 mg of the cryomilled powder resuspended in 750 µL of lysis buffer. Lysates were cleared by centrifugation at 16,000 × g at 4 °C for 15 minutes. Anti-FLAG magnetic agarose beads (Pierce) were washed on a magnet with 1 mL of ice-cooled IP buffer (50 mM Tris pH 7.4, 150 mM NaCl, 1 mM EDTA, 1 mM MgCl_2_) twice and resuspended to their original volume in IP buffer. Cleared lysates were incubated with 30 µL of FLAG beads on an end-over-end tube rotator at 10 rpm at 4 °C overnight. The supernatant was separated from the beads on a magnet. Beads were washed once with 700 µL of 0.05% NP-40 and 1x protease inhibitor cocktail in IP, once with 700 µL of 0.05% NP-40 in IP, and 3 times with 700 µL of IP. Proteins were eluted by incubation with 25 µL of 100 µg/ml 3xFLAG peptide (Sigma) in 0.05% RapiGest (Waters Corp) in IP buffer on an end-over-end tube rotator at 10 rpm at 4 °C overnight. The eluate was separated from the beads on a magnet. The elution step was then repeated with the same beads and fresh elution buffer at room temperature for 1 h. The eluates were combined and stored at −20 °C.

### Mass spectrometry sample preparation and data acquisition

Samples were prepared for Mass Spectrometry (MS) as described previously in the Filter-Aided Sample Preparation Protocol^61^ with the following modifications. Before transferring to an AcroPrep Advance 10k Omega plate, each eluate was resuspended in 100 µL of 8 M Urea (Thermo Scientific) dissolved in 50 mM ammonium bicarbonate (MP Biomedicals) and 5 mM TCEP (Aldrich), followed by centrifugation to dryness. During alkylation of cysteine residues, 10 mM chloroacetamide (Thermo Scientific) was used instead of 10 mM iodoacetamide. Wash steps prior to proteolysis were carried out with 200 μL of 20 mM ammonium bicarbonate (instead of 100 μL). Proteolysis was performed without the addition of lysyl endopeptidase. Proteolysis was quenched by addition of 10% trifluoroacetic acid (Thermo Scientific) to reach ≈ pH 2. Peptides were then purified over a BioPureSPE Mini 96-Well Plate (Nest Group). Briefly, C18 columns were activated by washing once with 400 µL of acetonitrile (Fisher Scientific) and twice with 400 µL of 0.1% formic acid (Thermo Scientific) in water solution, LC–MS grade (Thermo Scientific). Samples were loaded and centrifuged at 1500 X g for 2 min. The bound peptides were washed 3 times with 400 µL of 1% formic acid before elution with 150 µL of 50% acetonitrile in 0.1% trifluoroacetic acid. Peptides were vacuum dried and stored at −80 °C.

Samples were resuspended in 15 µl of 0.1% formic acid prior to loading for MS. The MS acquisition was performed as described^62^ with same MS parameters and LC configuration.

### Mass spectrometry data analysis and statistical analysis

Raw files were processed in FragPipe^63^ using the LFQ-MBR workflow with minor modifications. The files were searched against a combined database of protein sequences from ECOR1, ECOR3, ECOR15, ECOR21, ECOR22, ECOR66^56^, and MG1655 with duplicate entries removed and common contaminants added. Decoys were generated by pseudo-inversion. Protein and peptide FDR was fixed to 1% and MBR was disabled. Cysteine carbamylation was set as fixed modification while N-term acetylation, methionine oxidation and pyro-glu formation were set as variable modification with a maximum of 3 modifications per peptide.

MaxLFQ intensities were used for statistical analysis^64^. Each triplicate set of host-AG samples was quantile normalized against a triplicate set of samples from the same host expressing *orf74* as it did not produce any phenotypes in the AG screen. For quantile normalization, all proteins in each sample were ranked according to their MaxLFQ intensity. Next, the mean value of the intensities of proteins occupying a given rank was calculated, then each protein’s value was replaced with the mean value according to its rank. Fold changes were calculated by taking the log2 ratio of average normalized values between the test set and the control set. For each host-AG sample, all positively enriched proteins were ranked according to their fold change. For each AG, proteins were assigned “summed rank” values by calculating the sum of the ranks of each protein across all hosts where that AG was tested. P-values were calculated by student’s t-test on the normalized sets of replicates from the test and control samples. Top enriched candidates for each AG were selected based on their summed ranks and *p*-values.

### Allelic exchange for *wecA* deletion

The R6K-origin pKEK2201 vector (from Prof. Karl E. Klose) carrying the desired genetic modification (eg. a *wecA* deletion) flanked by 1000 bp homology arms was prepared by Gibson assembly, and delivered as a suicide vector into wild *E. coli* strains via conjugation from the WM6026 auxotrophic donor. Conjugation was performed using the same method used to deliver AGs, albeit with biparental instead of triparental mating. Successful transconjugants (containing a chromosomally integrated plasmid after the first homologous recombination event HR1) were selected by incubation on LB plates supplemented with kanamycin. The second recombination event HR2 to complete the allelic exchange was selected via *sacB*-mediated counterselection on LB plates supplemented with 300 mM sucrose after overnight growth of HR1 transconjugants. Successful allele exchange was verified by PCR, and by LPS electrophoresis to assay for the absence of O-antigen in the case of the *wecA* deletion.

### Co-immunoprecipitation of GalU and Gnarl3

Cells were collected from a saturated overnight culture of an *E. coli* TOP10 strain expressing an N-term GST-GalU fusion construct from a plasmid (pBAD promoter) and a C-term Gnarl3-3xFLAG fusion construct from a Tn7 single-copy chromosomal insertion (pLlacO-1 promoter). Cells were grown to mid-log phase (1:20 dilution of overnight cultures into 7 mL fresh LB with 100 µg/ml of ampicillin and 10 µg/ml gentamicin, grown at 37 °C for 1 h) and GST-GalU expression was induced by the addition of 0.1% w/v L-arabinose. Gnarl3-3xFLAG expression was induced by the addition of 1 mM IPTG one hour later, and cells were centrifuged at 8000 × g for 10 min after another hour. Whole cell lysates were prepared by resuspending cell pellets in 1 mL lysis buffer (50 mM Tris pH 7.4 at 4 °C, 150 mM NaCl, 10 mM EDTA, 1 mM MgCl_2,_ 0.5% NP40, 125 U/mL Benzonase, 1x protease inhibitor cocktail (Roche, complete ULTRA mini EDTA free), 0.5 mg/ml Lysozyme, 5 mM DTT), and sonicating with the samples continuously immersed in a circulating 4 °C water bath. Sonication was performed with a Bioruptor Pico bath sonicator for a total of 10 min (20 sec ON, 40 sec OFF, for a total of 30 cycles). Whole cell lysates were incubated with magnetic Glutathione agarose beads (Pierce) for 1 hour at room temperature with end-over-end rotation. Beads were then washed 2x in batch format with wash buffer 1 (50 mM Tris pH 7.4, 150 mM NaCl, 10 mM EDTA, 1 mM MgCl_2,_ 0.05% NP40) and then 3x with wash buffer 2 (same as wash buffer 1 but without detergent). Bound protein was eluted with 50 mM reduced glutathione prepared immediately before use in wash buffer 2 and adjusted for pH. Western blotting of cell lysates and eluates was performed as follows. Samples were heated at 95 °C for 5 min with 4x Laemmli buffer supplemented with ß-mercaptoethanol, resolved by SDS-PAGE, and transferred to PVDF membranes using the Bio-Rad Trans-Blot Turbo Transfer System according to the manufacturer’s instructions. Membranes were blocked for 1 hr in blocking buffer (5% w/v solution of non-fat dry milk in TBS-T: 1X Tris-buffered saline (20 mM Tris, 150 mM NaCl) supplemented with 0.1% Tween-20 detergent). Membranes were then incubated with a primary antibody (Rabbit anti-GST (Cell Signaling Technology 91G1) diluted 1:2500, or Mouse anti-FLAG (Sigma F1804) diluted 1:1000 in blocking buffer for 1 h, washed 3x with 25 mL TBS-T, then incubated with a secondary antibody (Anti-Rabbit HRP-linked IgG (Cell Signaling Technology 7074S) diluted 1:2000, or Anti-Mouse HRP-linked IgG (Invitrogen 62-6520) diluted 1:2000) for 1 hr, and washed 3x again with 25 mL TBS-T per wash. Membranes were developed using the Clarity Western ECL substrate kit (Bio-Rad) and imaged on an Azure bio-imaging system.

### Plaque assays with hypo-modified T4 phages

T4 5 hmC (lacking the glucosyl- modification) and T4 C (lacking both glucosyl- and hydroxymethyl- modifications) were received from the Bushman lab. T4 5hmC was propagated in DH10b, while T4 C was propagated in CR63. Prior to plating, T4 C was propagated in DH10b to ensure lack of modifications on its genome. Strains expressing various GmrSD enzymes were grown overnight in LB supplemented with 10 mM Mg^2+^ and 100ug/ml carbenicillin. 100ul of the overnight cultures was mixed with 5 ml of 0.4% top agar with or without arabinose inducer where indicated. Serial dilutions of propagated phages were then spotted before plates were allowed to dry and incubated at 37 °C overnight.

### Reporting summary

Further information on research design is available in the Nature Portfolio Reporting Summary linked to this article.

## Supplementary information

Supplementary Information

Description of Additional Supplementary Files

Supplementary Dataset 1

Supplementary Dataset 2

Supplementary Dataset 3

Supplementary Dataset 4

Supplementary Dataset 5

Supplementary Dataset 6

Reporting Summary

Transparent Peer review file

## Source data

Source data

## Data Availability

Supplementary Information is available for this paper. Mass Spectrometry data available at PRIDE https://www.ebi.ac.uk/pride/login with Username: reviewer_pxd038604@ebi.ac.uk and Password: 83OmeYpD. High-throughput sequencing data available at SRA with accession PRJNA952709 Correspondence and requests for materials should be addressed to Sukrit.Silas@gladstone.ucsf.edu, Joseph.Bondy-Denomy@ucsf.edu. Reprints and permissions information is available at www.nature.com/reprints. Source data are provided with this paper.

## References

[1] Doron, S. et al. Systematic discovery of antiphage defense systems in the microbial pangenome. *Science* **359**, eaar4120 (2018). 29371424 10.1126/science.aar4120 PMC6387622

[2] Gao, L. et al. Diverse enzymatic activities mediate antiviral immunity in prokaryotes. *Science* **369**, 1077–1084 (2020). 32855333 10.1126/science.aba0372 PMC7985843

[3] Millman, A. et al. An expanded arsenal of immune systems that protect bacteria from phages. *Cell host microbe* **30**, 1556–1569 e1555 (2022). 36302390 10.1016/j.chom.2022.09.017

[4] Vassallo, C. N., Doering, C. R., Littlehale, M. L., Teodoro, G. I. C. & Laub, M. T. A functional selection reveals previously undetected anti-phage defence systems in the E. coli pangenome. *Nat. Microbiol* **7**, 1568–1579 (2022). 36123438 10.1038/s41564-022-01219-4 PMC9519451

[5] Rousset, F. et al. Phages and their satellites encode hotspots of antiviral systems. *Cell host microbe* **30**, 740–753.e745 (2022). 35316646 10.1016/j.chom.2022.02.018 PMC9122126

[6] Tesson, F. & Bernheim, A. Synergy and regulation of antiphage systems: toward the existence of a bacterial immune system?. *Curr. Opin. Microbiol.* **71**, 102238 (2023). 36423502 10.1016/j.mib.2022.102238

[7] Mitarai, N., Marantos, A. & Sneppen, K. Sustainable diversity of phage-bacteria systems. *Curr. Opin. Syst. Biol.* **35**, 100468 (2023).

[8] Rostøl, J. T. & Marraffini, L. Ph)ighting Phages: How Bacteria Resist Their Parasites. *Cell host microbe* **25**, 184–194 (2019). 30763533 10.1016/j.chom.2019.01.009 PMC6383810

[9] Guillemet, M. et al. Competition and coevolution drive the evolution and the diversification of CRISPR immunity. *Nat. Ecol. Evol.* **6**, 1480–1488 (2022). 35970864 10.1038/s41559-022-01841-9

[10] Koonin, E. V., Makarova, K. S. & Wolf, Y. I. Evolutionary Genomics of Defense Systems in Archaea and Bacteria. *Annu. Rev. Microbiol.* **71**, 233–261 (2017). 28657885 10.1146/annurev-micro-090816-093830 PMC5898197

[11] Makarova, K. S., Wolf, Y. I. & Koonin, E. V. In *Crispr* 13-38 (2022).

[12] Maxwell, K. L. The Anti-CRISPR Story: A Battle for Survival. *Mol. cell* **68**, 8–14 (2017). 28985512 10.1016/j.molcel.2017.09.002

[13] Tesson, F. et al. Systematic and quantitative view of the antiviral arsenal of prokaryotes. *Nat. Commun.* **13**, 2561 (2022). 35538097 10.1038/s41467-022-30269-9 PMC9090908

[14] Silas, S. et al. Activation of bacterial programmed cell death by phage inhibitors of host immunity. *Mol. cell* **85**, 1838–1851.e1810 (2025). 40315827 10.1016/j.molcel.2025.04.010 PMC12088041

[15] Liu, B. et al. Structure and genetics of Escherichia coli O antigens. *FEMS Microbiol. Rev.* **44**, 655–683 (2020). 31778182 10.1093/femsre/fuz028 PMC7685785

[16] Whitfield, C., Williams, D. M. & Kelly, S. D. Lipopolysaccharide O-antigens-bacterial glycans made to measure. *J. Biol. Chem.* **295**, 10593–10609 (2020). 32424042 10.1074/jbc.REV120.009402 PMC7397119

[17] Cunneen, M. M. & Reeves, P. R. In *Bacterial Lipopolysaccharides: Structure, Chemical Synthesis, Biogenesis and Interaction with Host Cells* (eds Yuriy A. Knirel & Miguel A. Valvano) 339-370 (Springer Vienna, 2011).

[18] Lindberg, A. A. Bacteriophage Receptors. *Annu. Rev. Microbiol.* **27**, 205–241 (1973). 4584686 10.1146/annurev.mi.27.100173.001225

[19] Heller, K. & Braun, V. Polymannose O-antigens of Escherichia coli, the binding sites for the reversible adsorption of bacteriophage T5+ via the L-shaped tail fibers. *J. Virol.* **41**, 222–227 (1982). 7045389 10.1128/jvi.41.1.222-227.1982 PMC256742

[20] Uetake, H., Luria, S. E. & Burrous, J. W. Conversion of somatic antigens in Salmonella by phage infection leading to lysis or lysogeny. *Virology* **5**, 68–91 (1958). 13519750 10.1016/0042-6822(58)90006-0

[21] Allison, G. E. & Verma, N. K. Serotype-converting bacteriophages and O-antigen modification in Shigella flexneri. *Trends Microbiol* **8**, 17–23 (2000). 10637639 10.1016/s0966-842x(99)01646-7

[22] Vander Byl, C. & Kropinski, A. M. Sequence of the genome of Salmonella bacteriophage P22. *J. Bacteriol.* **182**, 6472–6481 (2000). 11053393 10.1128/jb.182.22.6472-6481.2000 PMC94795

[23] Kulikov, E. E. et al. Equine Intestinal O-Seroconverting Temperate Coliphage Hf4s: Genomic and Biological Characterization. *Appl. Environ. Microbiol.* **87**, e0112421 (2021). 34406832 10.1128/AEM.01124-21 PMC8516047

[24] Newton, G. J. et al. Three-component-mediated serotype conversion in Pseudomonas aeruginosa by bacteriophage D3. *Mol. Microbiol* **39**, 1237–1247 (2001). 11251840 10.1111/j.1365-2958.2001.02311.x

[25] Losick, R. Isolation of a trypsin-sensitive inhibitor of O-antigen synthesis involved in lysogenic conversion by bacteriophage epsilon-15. *J. Mol. Biol.* **42**, 237–246 (1969). 5803297 10.1016/0022-2836(69)90040-0

[26] Kropinski, A. M. et al. The genome of epsilon15, a serotype-converting, Group E1 Salmonella enterica-specific bacteriophage. *Virology* **369**, 234–244 (2007). 17825342 10.1016/j.virol.2007.07.027 PMC2698709

[27] Repoila, F., Tetart, F., Bouet, J. Y. & Krisch, H. M. Genomic polymorphism in the T-even bacteriophages. *EMBO J.* **13**, 4181–4192 (1994). 8076614 10.1002/j.1460-2075.1994.tb06736.x PMC395341

[28] Black, L. W. & Abremski, K. Restriction of phage T4 internal protein I mutants by a strain of Escherichia coli. *Virology* **60**, 180–191 (1974). 4601629 10.1016/0042-6822(74)90375-4

[29] Bair, C. L., Rifat, D. & Black, L. W. Exclusion of glucosyl-hydroxymethylcytosine DNA containing bacteriophages is overcome by the injected protein inhibitor IPI*. *J. Mol. Biol.* **366**, 779–789 (2007). 17188711 10.1016/j.jmb.2006.11.049 PMC1868451

[30] Ochman, H. & Selander, R. K. Standard reference strains of Escherichia coli from natural populations. *J. Bacteriol.* **157**, 690–693 (1984). 6363394 10.1128/jb.157.2.690-693.1984 PMC215307

[31] Mutalik, V. K. et al. High-throughput mapping of the phage resistance landscape in E. coli. *PLoS Biol.* **18**, e3000877 (2020). 33048924 10.1371/journal.pbio.3000877 PMC7553319

[32] Loenen, W. A. & Murray, N. E. Modification enhancement by the restriction alleviation protein (Ral) of bacteriophage lambda. *J. Mol. Biol.* **190**, 11–22 (1986). 3023633 10.1016/0022-2836(86)90071-9

[33] Gao, Y. et al. Structural insights into assembly, operation and inhibition of a type I restriction-modification system. *Nat. Microbiol* **5**, 1107–1118 (2020). 32483229 10.1038/s41564-020-0731-z

[34] Murphy, K. C. Bacteriophage P22 Abc2 protein binds to RecC increases the 5’ strand nicking activity of RecBCD and together with lambda bet, promotes Chi-independent recombination. *J. Mol. Biol.* **296**, 385–401 (2000). 10669596 10.1006/jmbi.1999.3486

[35] Dougherty, P. E. et al. Persistent virulent phages exist in bacterial isolates. *bioRxiv*, 2024.2012.2031.630880 10.1101/2024.12.31.630880 (2025).

[36] Weissborn, A. C., Liu, Q., Rumley, M. K. & Kennedy, E. P. UTP: alpha-D-glucose-1-phosphate uridylyltransferase of Escherichia coli: isolation and DNA sequence of the galU gene and purification of the enzyme. *J. Bacteriol.* **176**, 2611–2618 (1994). 8169209 10.1128/jb.176.9.2611-2618.1994 PMC205399

[37] Genevaux, P., Bauda, P., DuBow, M. S. & Oudega, B. Identification of Tn10 insertions in the rfaG, rfaP, and galU genes involved in lipopolysaccharide core biosynthesis that affect Escherichia coli adhesion. *Arch. Microbiol* **172**, 1–8 (1999). 10398745 10.1007/s002030050732

[38] Marolda, C. L. & Valvano, M. A. The GalF protein of Escherichia coli is not a UDP-glucose pyrophosphorylase but interacts with the GalU protein possibly to regulate cellular levels of UDP-glucose. *Mol. Microbiol* **22**, 827–840 (1996). 8971705 10.1046/j.1365-2958.1996.01531.x

[39] Pierson, D. E. & Carlson, S. Identification of the galE gene and a galE homolog and characterization of their roles in the biosynthesis of lipopolysaccharide in a serotype O:8 strain of Yersinia enterocolitica. *J. Bacteriol.* **178**, 5916–5924 (1996). 8830687 10.1128/jb.178.20.5916-5924.1996 PMC178447

[40] Fratamico, P. M., Briggs, C. E., Needle, D., Chen, C. Y. & DebRoy, C. Sequence of the Escherichia coli O121 O-antigen gene cluster and detection of enterohemorrhagic E. coli O121 by PCR amplification of the wzx and wzy genes. *J. Clin. Microbiol* **41**, 3379–3383 (2003). 12843098 10.1128/JCM.41.7.3379-3383.2003 PMC165269

[41] Machnicka, M. A., Kaminska, K. H., Dunin-Horkawicz, S. & Bujnicki, J. M. Phylogenomics and sequence-structure-function relationships in the GmrSD family of Type IV restriction enzymes. *BMC Bioinforma.* **16**, 336 (2015). 10.1186/s12859-015-0773-z PMC4619093 26493560

[42] Lehrer, J., Vigeant, K. A., Tatar, L. D. & Valvano, M. A. Functional characterization and membrane topology of Escherichia coli WecA, a sugar-phosphate transferase initiating the biosynthesis of enterobacterial common antigen and O-antigen lipopolysaccharide. *J. Bacteriol.* **189**, 2618–2628 (2007). 17237164 10.1128/JB.01905-06 PMC1855806

[43] Gordeeva, J. et al. BREX system of Escherichia coli distinguishes self from non-self by methylation of a specific DNA site. *Nucleic acids Res.* **47**, 253–265 (2019). 30418590 10.1093/nar/gky1125 PMC6326788

[44] Picton, D. M. et al. The phage defence island of a multidrug resistant plasmid uses both BREX and type IV restriction for complementary protection from viruses. *Nucleic acids Res.* **49**, 11257–11273 (2021). 34657954 10.1093/nar/gkab906 PMC8565348

[45] Isaev, A. et al. Phage T7 DNA mimic protein Ocr is a potent inhibitor of BREX defence. *Nucleic acids Res.* **48**, 5397–5406 (2020). 32338761 10.1093/nar/gkaa290 PMC7261183

[46] Srikant, S., Guegler, C. K. & Laub, M. T. The evolution of a counter-defense mechanism in a virus constrains its host range. *eLife* **11**, e79549 (2022). 35924892 10.7554/eLife.79549 PMC9391042

[47] Bryson, A. L. et al. Covalent Modification of Bacteriophage T4 DNA Inhibits CRISPR-Cas9. *mBio* **6**, e00648 (2015). 26081634 10.1128/mBio.00648-15 PMC4471564

[48] Shmakov, S. A. et al. Widespread CRISPR-derived RNA regulatory elements in CRISPR-Cas systems. *Nucleic acids Res.* **51**, 8150–8168 (2023). 37283088 10.1093/nar/gkad495 PMC10450183

[49] Steinegger, M. & Soding, J. MMseqs2 enables sensitive protein sequence searching for the analysis of massive data sets. *Nat. Biotechnol.* **35**, 1026–1028 (2017). 29035372 10.1038/nbt.3988

[50] Labrie, S. J., Samson, J. E. & Moineau, S. Bacteriophage resistance mechanisms. *Nat. Rev. Microbiol.* **8**, 317–327 (2010). 20348932 10.1038/nrmicro2315

[51] Franklin, N. C. Mutation in gal U gene of E. coli blocks phage P1 infection. *Virology* **38**, 189–191 (1969). 4891220 10.1016/0042-6822(69)90144-5

[52] Young, F. E. Requirement of glucosylated teichoic acid for adsorption of phage in Bacillus subtilis 168. *Proc. Natl Acad. Sci. USA* **58**, 2377–2384 (1967). 4969329 10.1073/pnas.58.6.2377 PMC223846

[53] Davies, M. R., Broadbent, S. E., Harris, S. R., Thomson, N. R. & van der Woude, M. W. Horizontally acquired glycosyltransferase operons drive salmonellae lipopolysaccharide diversity. *PLoS Genet.* **9**, e1003568 (2013). 23818865 10.1371/journal.pgen.1003568 PMC3688519

[54] Bondy-Denomy, J. et al. Prophages mediate defense against phage infection through diverse mechanisms. *ISME J.* **10**, 2854–2866 (2016). 27258950 10.1038/ismej.2016.79 PMC5148200

[55] Peters, J. M. et al. Enabling genetic analysis of diverse bacteria with Mobile-CRISPRi. *Nat. Microbiol* **4**, 244–250 (2019). 30617347 10.1038/s41564-018-0327-z PMC6424567

[56] Patel, I. R. et al. Draft Genome Sequences of the Escherichia coli Reference (ECOR) Collection. *Microbiol Resour. Announc* **7**, e01133–18 (2018). 30533715 10.1128/MRA.01133-18 PMC6256646

[57] Lu, M. J. & Henning, U. Superinfection exclusion by T-even-type coliphages. *Trends Microbiol* **2**, 137–139 (1994). 8012757 10.1016/0966-842x(94)90601-7

[58] Uc-Mass, A. et al. An orthologue of the cor gene is involved in the exclusion of temperate lambdoid phages. Evidence that Cor inactivates FhuA receptor functions. *Virology* **329**, 425–433 (2004). 15518820 10.1016/j.virol.2004.09.005

[59] Wetmore, K. M. et al. Rapid quantification of mutant fitness in diverse bacteria by sequencing randomly bar-coded transposons. *mBio* **6**, e00306–e00315 (2015). 25968644 10.1128/mBio.00306-15 PMC4436071

[60] Phillips, E. O. N., Giovinazzi, S., Menz, S. L., Son, Y. & Gunjan, A. Preparation of Cell Extracts by Cryogrinding in an Automated Freezer Mill. *J Vis Exp*10.3791/61164 (2021). 10.3791/61164 33586710

[61] Fossati, A. et al. System-Wide Profiling of Protein Complexes Via Size Exclusion Chromatography-Mass Spectrometry (SEC-MS). *Methods Mol. Biol.* **2259**, 269–294 (2021). 33687722 10.1007/978-1-0716-1178-4_18

[62] Gordon, D. E. et al. A SARS-CoV-2 protein interaction map reveals targets for drug repurposing. *Nature* **583**, 459–468 (2020). 32353859 10.1038/s41586-020-2286-9 PMC7431030

[63] Kong, A. T., Leprevost, F. V., Avtonomov, D. M., Mellacheruvu, D. & Nesvizhskii, A. I. MSFragger: ultrafast and comprehensive peptide identification in mass spectrometry-based proteomics. *Nat. methods* **14**, 513–520 (2017). 28394336 10.1038/nmeth.4256 PMC5409104

[64] Cox, J. et al. Accurate proteome-wide label-free quantification by delayed normalization and maximal peptide ratio extraction, termed MaxLFQ. *Mol. Cell Proteom.* **13**, 2513–2526 (2014). 10.1074/mcp.M113.031591 PMC4159666 24942700

