# Supplementary material for: Anti-restriction functions of injected phage proteins revealed by peeling back layers of bacterial immunity
Source: Nat Commun. 2025 Aug 22;16:7828. doi: 10.1038/s41467-025-63056-3 (PMC12373910; doi:10.1038/s41467-025-63056-3)
Supplement: Supplementary file 1 — Supplementary Information [file 41467_2025_63056_MOESM1_ESM.pdf]

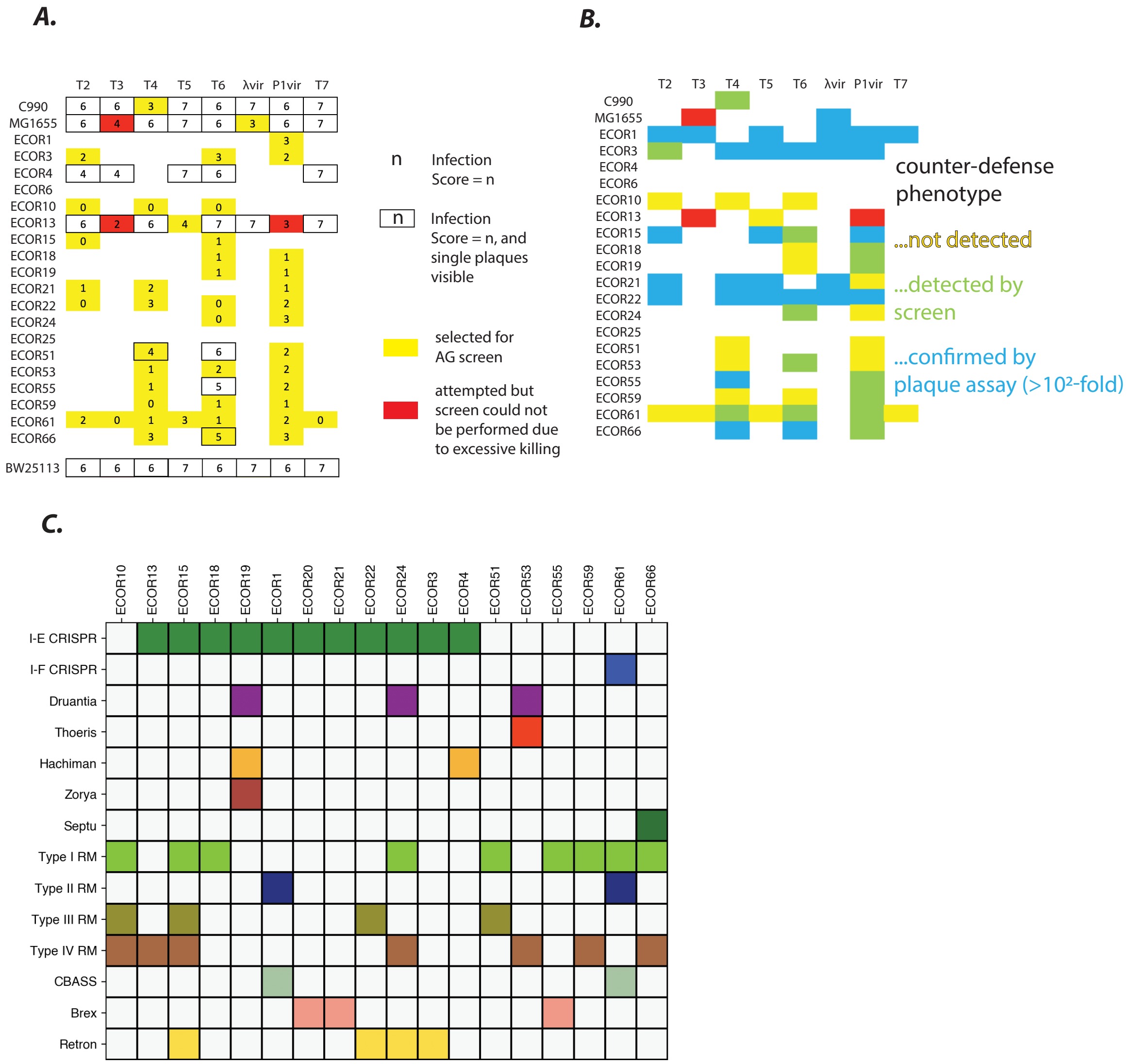

Supplementary Figure 1. Phage-host combinations included in AG screens (A) Phage susceptibility of select wild ECOR strains. Numbers in boxes are log10-transformed infection scores (7 = 10^6-fold higher plaquing than 1; 0 = very faint clearings produced by undiluted phage lysate; empty box = host completely immune). Borders indicate that individual plaques were visible in that phage-host combination. Combinations with attenuated phage infection (relative to BW25113) that were selected for the AG screen are in yellow. Combinations with attenuated infection that were attempted but yielded no survivors in liquid infection experiments (perhaps due to Abi phenotypes) are in red. **(B)** Phage-host combinations where counter-defense phenotypes were confirmed for any AGs. Red denotes combinations where the screen could not be completed (as in (A)). Yellow: combinations that were tested where none of the 196 AGs produced counter-defense effects. Green: combinations where phage-sensitizing effects could not be verified by plaque assays. Blue: combinations where counter-defense phenotypes were confirmed by plaque assay (at least 100X higher EOP upon AG expression). **(C)** Defense repertoire of selected ECOR strains. Computational prediction of the presence or absence of various defense systems in ECOR strains used in the AG screen. Predictions were generated using the ISLAND software suite[55].

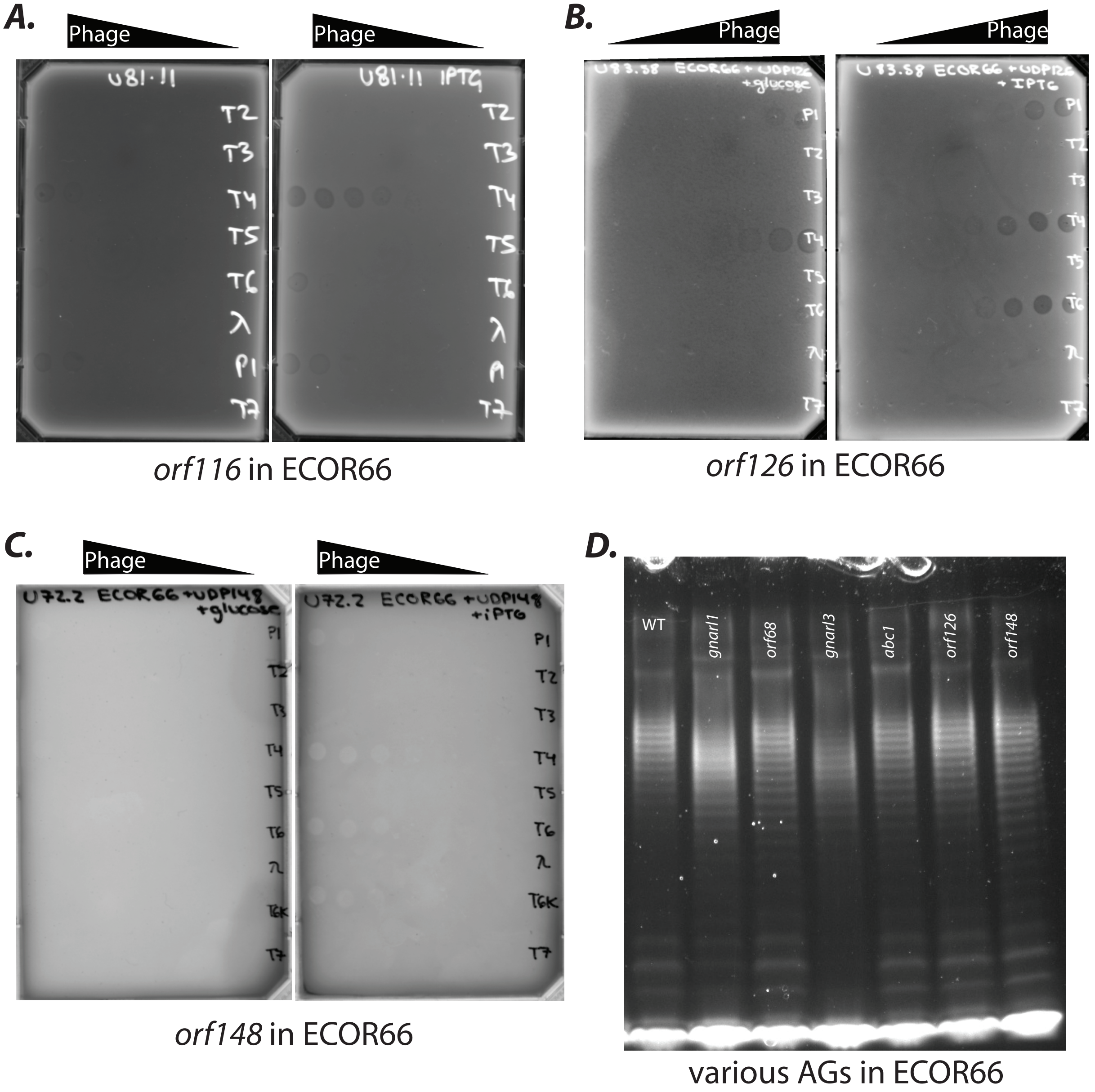

Supplementary Figure 2. Plaque assays and LPS electrophoresis with ECOR66 expressing *orfs 116, 126, 148*. (A-C) Representative images from two replicate plaque assays (performed by two different experimenters) with ECOR66 expressing *orfs 116* (*abc1*), 126, and 148. Left panel ("glucose") shows plaque assays with AGs repressed, right panel ("IPTG") shows plaque assays with AGs expressed. **(D)** LPS electrophoresis of ECOR66 expressing AGs that produced a phage-sensitizing effect against any phage.

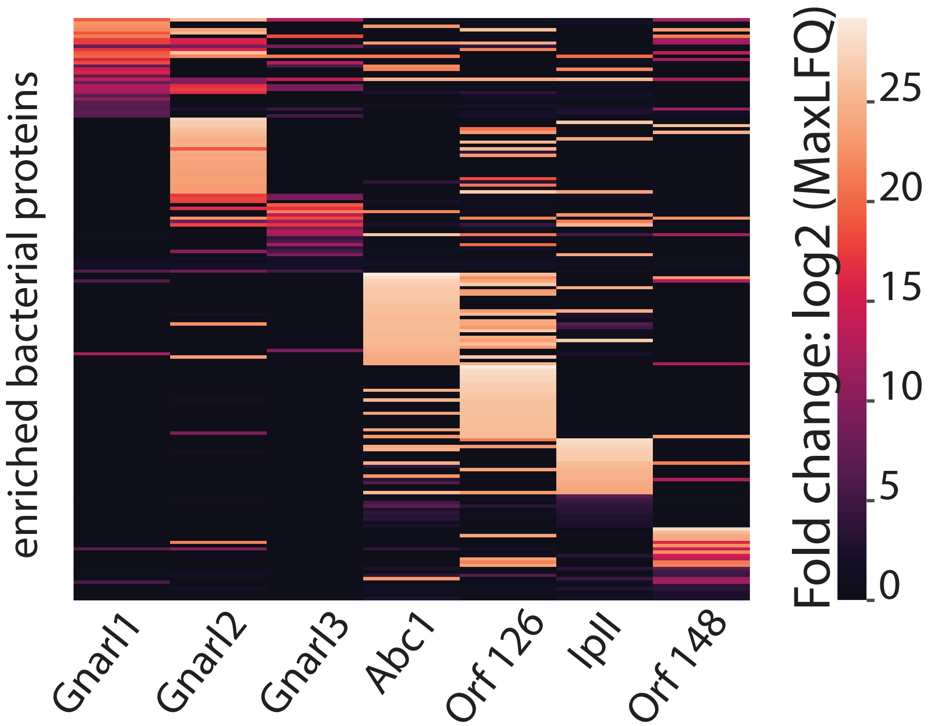

Supplementary Figure 3. Log2-transformed protein representation scores (MaxLFQ) for the top 30 enriched host proteins upon expression of FLAG-tagged AG-products. Named AGs are *orf48:gnarl1, orf63:gnarl2, orf92:gnarl3, orf116:abc1, orf143:ip2*. Fold changes were calculated by taking the log2 ratio of average normalized MaxLFQ values between the test set and the control set in triplicate experiments (see Supplementary Data 4).

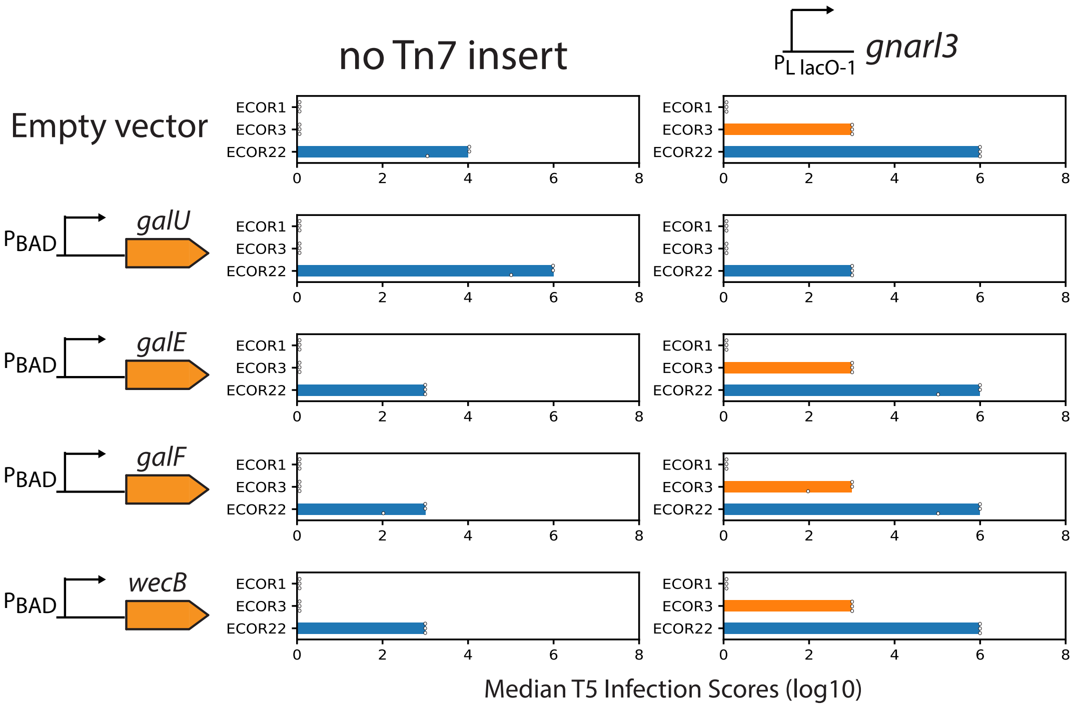

Supplementary Figure 4. Over-expression of *galU* reverses T4 sensitization by *gnarl3*. Log10-transformed T4 infection scores in wild hosts ECOR1 (green), ECOR3 (orange), ECOR22 (blue) with or without UDP-glucose biosynthesis pathway genes *galU, galE, galF,* and Enterobacterial Common Antigen (ECA) precursor *wecB* cloned onto a plasmid and overexpressed. Infection scores from triplicate plaque assays are depicted by circles.

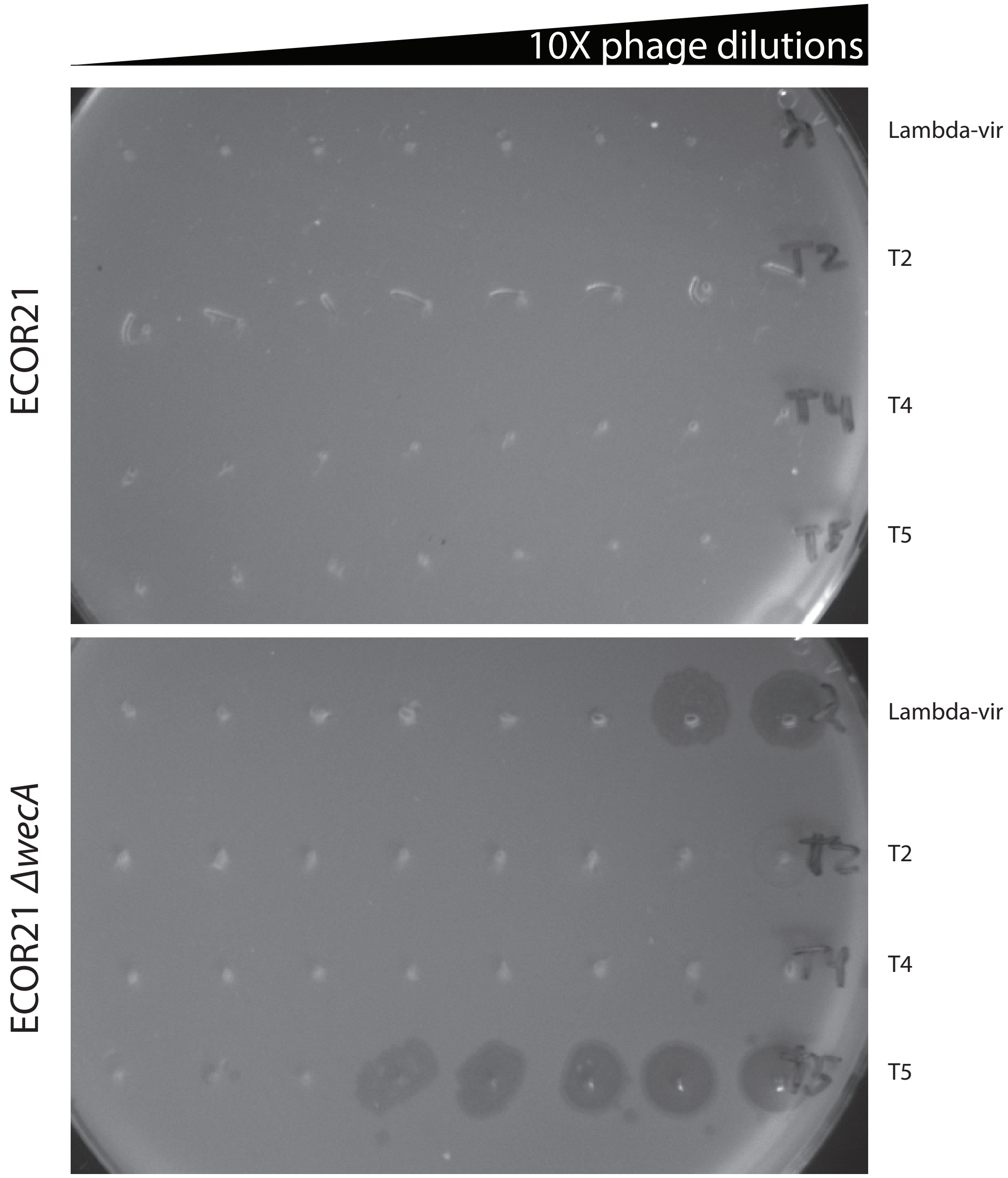

Supplementary Figure 5. O-antigen removal sensitizes ECOR21 to various phages. Effect of O-antigen removal via the deletion of *wecA* on susceptibility of ECOR21 to phages in Figure 3a. Plaque assays with ECOR21 or ECOR21Δ*wecA* with 10-fold serial dillutions of indicated phages.

A.

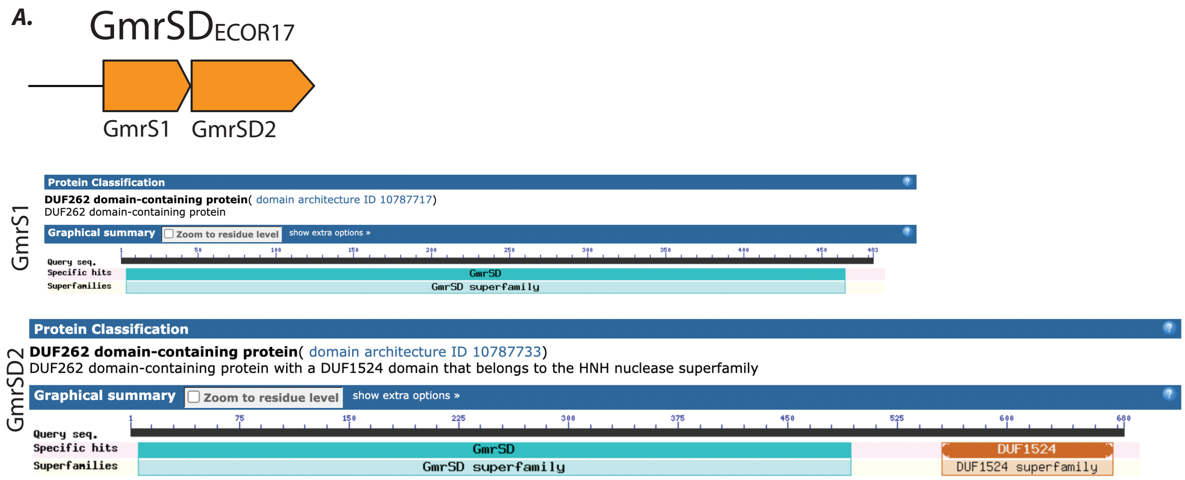

B.

```
GmrS1      MESINNQVQSVRQL-LAIPQLAIPSYQRPYRWGSKNISDLFTDLATHQDK-------SAY      52
GmrSD2     MNDAGVRPFSIRALLEDRARYLVPMYQRNYAWGEGEITQLLQDVLDYQQKLVSGKGPQTY      60
           *:. . :  *:* *     :  :* *** * **. :*::*: *:  :*:*       .:*

GmrS1      RLGSVVFHQHTDSEQGEMLDIVDGQQRTLTLMLTVKALIEVLDNENRSGKEKSIRFQRQD      112
GmrSD2     YIGTLVVFARD----DGSFEVIDGQQRFTTLSLLANWLKHHAIDS----VD-MSWYQTIN      111
            :*::*.. :     .  ::::*****  ** * .: * .   :.     :    :*  :

GmrS1      LREQLQVLRGPIDAFM------QRQRFPSRDSEFNLR--RNYLELRRLVARPEF----DE      160
GmrSD2     L----AFESRPISSHTFERLWQGVAPYDLRGSTFNEGLVNGFELIGKAMADLGLVGAKLT      167
           *     .   **.:.           :  *.* **    ..:  : : :*   :

GmrS1      QQIDFLLNRCQVVCFVLQDISEAFQFFDSQNARGRDLAPHDLLKAFHLREFAGHEANLKA      220
GmrSD2     AFCDYLFKHVQISRIEVPKDTDLNHFFEAMNNRGEQLEKHEVIKARLMETLNQIPEEEHR      227
             *:*::: *:  : : . ::  :**:: * **.:*  *:::**  :. :     : :

GmrS1      -----EAVAHWERLPSDELANLFALYLYRVRQWAEGKSARYFGKGEVDLFKGVNLD----      271
GmrSD2     RQSIHILTRVWDACANME-----R---YIQYGFTPQERHRLFGESDWGQFVPRDFAHLLD      279
                  .  *:   . *         *    ::  :  * **:.: . *   ::

GmrS1      RVGHYPYVESLRIAHHFVDEY-----NSQYQRKIDGQYMTFPFHLDQMIINGRRFFEMAE      326
GmrSD2     LLGSPNTADAADKSRAAAGSQGRTLLAILQDDKLDGEQT-----VEEESAGSERFNSVIN      334
            :*    .::   ::  ...           : *:**:       :::   ...** .: :

GmrS1      YYQTRVAAIVAEESDSGKAQSATLLGETLTP-MASKVLSTLGSYERRHRTGDRYVRAMFD      385
GmrSD2     FSNFLLHVLRLVSCDPGDTEGVPLDDKQLVDQFELRVIRQPDP----VAAVQRFIYGLLK      390
           : :  : .:   ..* *.::.. * .: *.  :  :*:   .       : :*:: .::.

GmrS1      CALIF---YI-DKFGSQGLSLAIEKCFIWAYRCRIRQQVVQLATMDNYVLEHNLIRAIRD      441
GmrSD2     SKYLFDQFIIKREFADGKDSWSL-KRLHWYSEKS----VSYI------------------      427
           .  :*    *  :*..   * :: * : *  .      *  :

GmrS1      ARLPGDLHGFPLPSMSSRENKNNRNGAEDELVGLFRE-----------------------      478
GmrSD2     -----------NTFDKDENENGFSGVNRRVLMLLSAFHVSTPTLVYKHWLNGALRYLFD      475
                        ::.. **:*. .*.: .:: *:

GmrS1      ----------------------------------MKYYE---------------------      483
GmrSD2     NCHPDQPVDAGAYLSYLESQARRFVFQRFLAPGEGASYYQMLYLDNALLPAINVDESWHK      535
                                            .**:

GmrS1      ------------------------------------------------------------      483
GmrSD2     VITSKLRFGHIENNFVFNFLDYLLWVRDRNSDKVAGSFEFTFRSSVEHFSPQHPMDGYKP      595


GmrS1      ------------------------------------------------------------      483
GmrSD2     VEQSALHSFGNLCLISHSKNSRLSNFQPQQKQEHFEASLANNQTDSLKLLAMIRLMKDKG      655


GmrS1      -------------------------   483
GmrSD2     RWLEDEIAVHQQDMLQVLLSNQIQQ   680
```

Supplementary Figure 6. Domain analysis and alignment of $GmrS_1$ and $GmrSD_2$ components of $GmrSD_{ECOR17}$. (A) Protein domains analyzed by NCBI CD-search webserver. **(B)** Alignment of $GmrS_1$ and $GmrSD_2$ protein sequences.

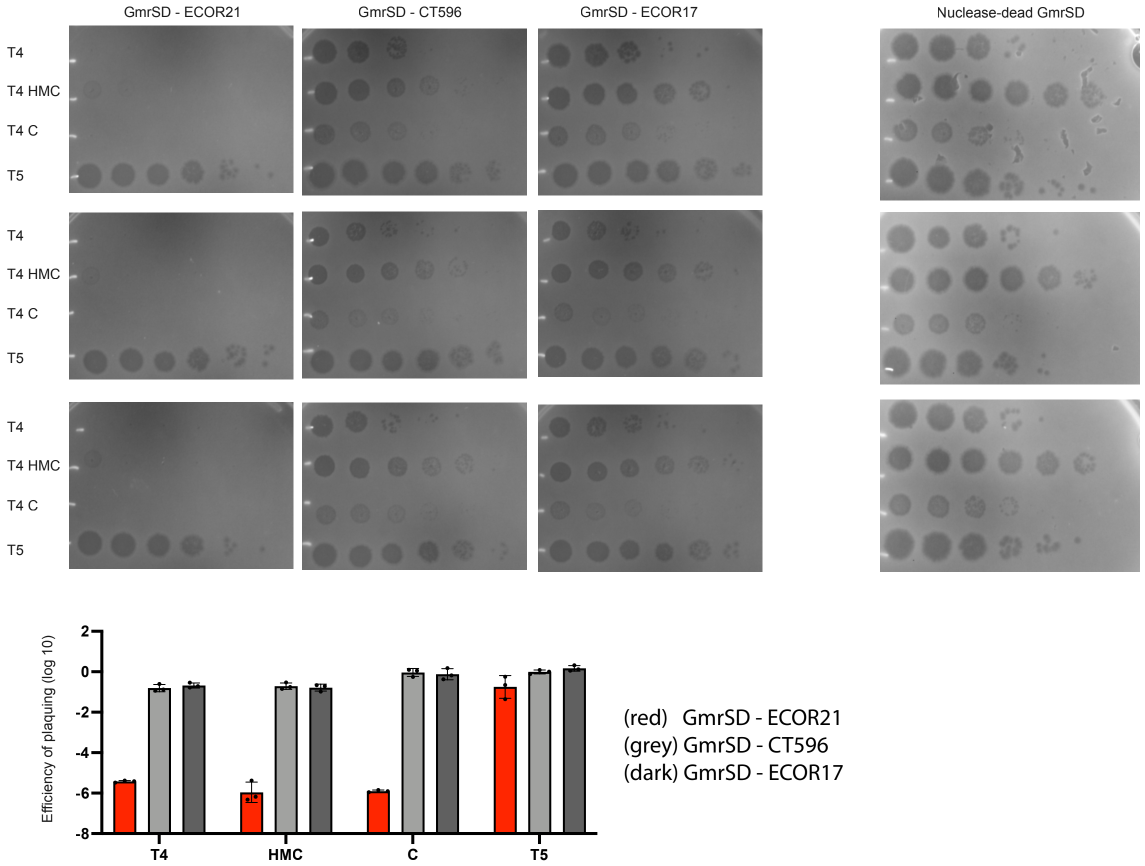

Supplementary Figure 7. Substrate specificity of GmrSD variants. Representative images and bar-graph quantification of triplicate plaque assays with TOP10 *E. coli* expressing GmrSD variants from ECOR21, CT596, and ECOR17 challenged by (1) T4: wildtype T4 phage with g-hmC- (glucosyl-hydroymethylcytosine-) modified DNA, (2) T4 HMC: non-glucosylated T4 with 5hmC- (hydroxymethylcytosine-) modified DNA, and (3) T4 C: T4 with unmodified DNA. None of the GmrSD constructs were overexpressed from the pBAD promoter, as in Figure 5. $GmrSD_{ECOR21}$ is expressed from its native promoter embedded in BREX locus, and $GmrSD_{ECOR17}$ and $GmrSD_{CT596}$ could only be tested under "leaky" expression conditions due to their gross cellular toxicity. "Nuclease dead" variant contains active-site mutations in $GmrSD_{ECOR21}$.

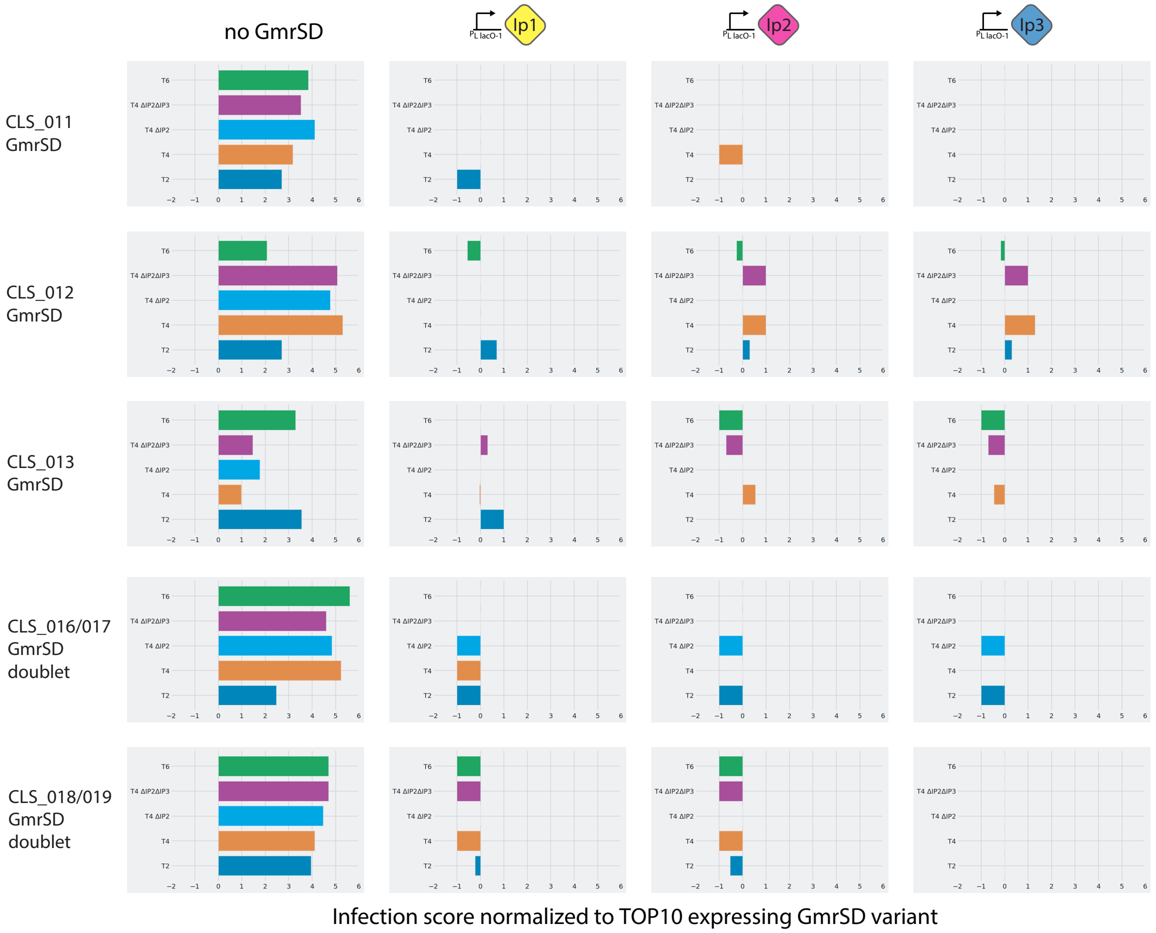

Supplementary Figure 8. Injected proteins (*ip*) from T4 phage block specific GmrSD variants. Plaque assays to measure anti-phage activity of other common GmrSD variants with heterologous expression of Ip1-3. *ip* genes are expressed from single-copy chromosomal insertions in TOP10 *E. coli*. All plasmid-encoded GmrSD genes were tested with 0.05-0.1% L-arabinose induction from a pBAD vector. Bar graphs show normalized log10 infection scores relative to strain with the respective GmrSD variant but no *ip*. Hence, the first ("no GmrSD") column shows infection score of the phage in TOP10 without GmrSD, *ip* (i.e. fully permissive infection), relative to a TOP10 strain with the respective GmrSD only. Subsequent columns are comparing TOP10 with the indicated GmrSD + *ip* combination, relative to GmrSD only. Higher normalized infection scores imply greater phage success.

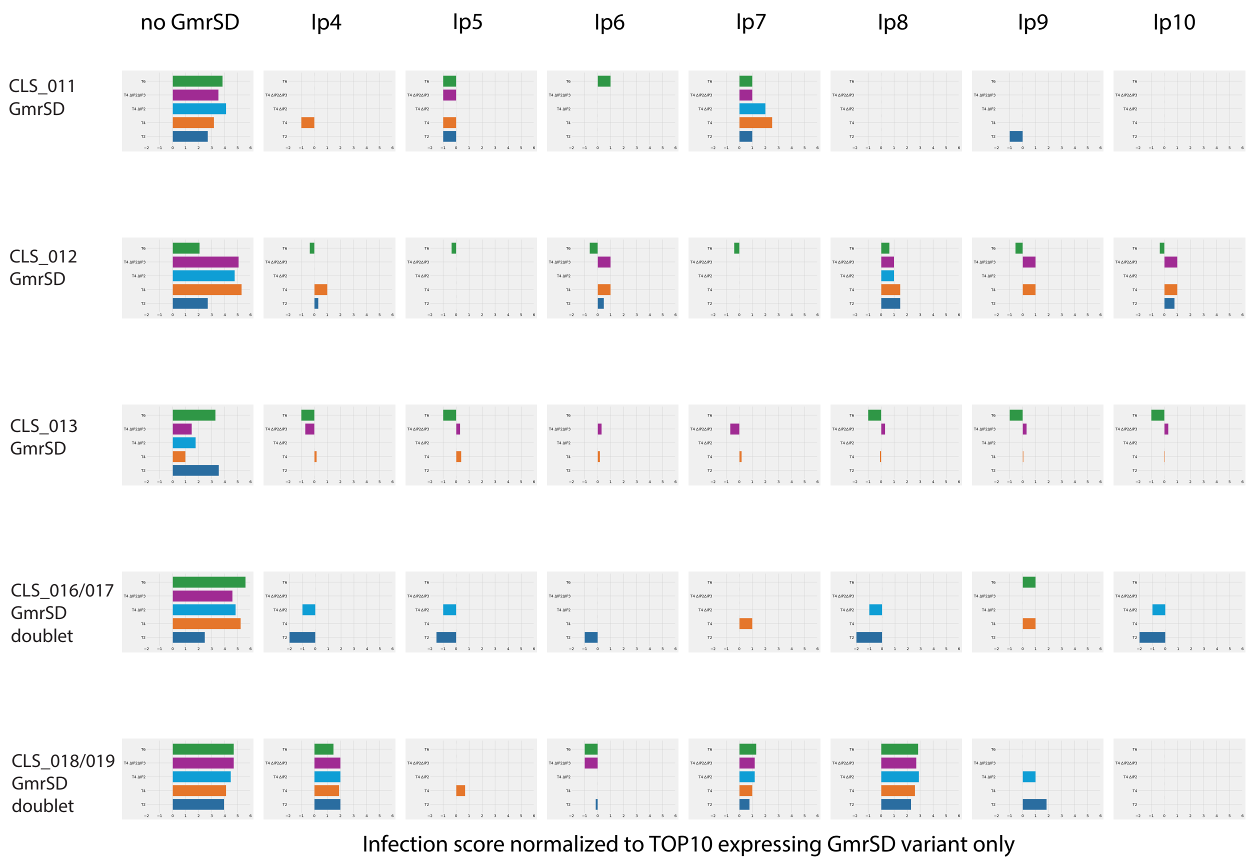

Supplementary Figure 9. Other injected proteins (*ip*) from T4-like phages tested against GmrSD variants. As in Supplementary Figure 8, but with *ip4-10*. Apparent interactions (e.g. *ip7* with CLS_011 or *ip8* with the CLS_018/019 doublet GmrSD system could not be reproduced in follow-up triplicate plaque assays.

Supplementary Table 1. Critical reagents and materials

REAGENT or RESOURCE	SOURCE	IDENTIFIER
**Antibodies**		
Rabbit anti-GST	Cell Signaling Technology	91G1
Mouse anti-FLAG	Sigma	F1804
Anti-Rabbit HRP-linked IgG	Cell Signaling Technology	7074S
Anti-Mouse HRP-linked IgG	Invitrogen	62-6520
Mouse anti-*E. coli RNAPß*	Biolegend	663903
**Bacterial and virus strains**		
ECOR 1-72	STEC Center	N/A
E. coli MG1655	Carol Gross' Lab Stocks	N/A
E. coli C990	Ry Young	N/A
E. coli DH5alpha	NEB	C2987H
E. coli TOP10	Thermo Fisher Scientific	C404010
E. coli BW25113	Vivek Mutalik	N/A
E. coli BW25141	Jason Peters	N/A
E. coli WM6026	Jason Peters	N/A
E. coli DHT1	Scot Oullette	N/A
T2-T7 phages	Vivek Mutalik	N/A
Lambda-vir phage	Ry Young	N/A
**Chemicals, peptides, and recombinant proteins**		
IPTG isopropyl-b-D-thiogalactopyranoside	VWR	76800-062
L-arabinose	VWR	TCA0515
D-Glucose	VWR	97061-166
2,6-diaminopimelic acid	Sigma Aldrich	33240
X-α-Gal, 5-Bromo-4-chloro-3-indolyl-α-D-galactopyranoside	Millipore Sigma	16555
Benzonase nuclease, recombinant	Sigma Aldrich	E1014
Lysozyme, chicken egg white	VWR	AAJ60701-03
cOmplete™ ULTRA Tablets, Mini, EDTA-free, EASYpack Protease Inhibitor Cocktail	Sigma Aldrich	5892791001
Pierce™ Glutathione Magnetic Agarose Beads	Thermo Fisher Scientific	78602
L-glutathione, reduced	Millipore Sigma	G4251
**Critical commercial assays**		
Genomic DNA Clean and Concentrator Kit	Zymo Research	D4065
Gel DNA recovery Kit	Zymo Research	D4008
Plasmid miniprep Kit - Classic	Zymo Research	D4054
Illumina DNA prep kit	Illumina	20060060
NEB Ultra II FS DNA Library Prep Kit	NEB	E7805L

2X Gibson assembly master mix	NEB	E2611L
NEBuilder HiFi DNA assembly master mix	NEB	E2621L
1x dsDNA HS assay kit	Thermo Fisher Scientific	Q33231
Luna® Universal One-Step RT-qPCR Kit	NEB	E3005L
AMpure XP beads	Beckman Coulter	A63881
**Oligonucleotides**		
JSW-SS-170 (Ns are mixed bases) CGACGCTCTTCCGATCTNNNNNTGATGTCGTTGTTGCCATCG		
JSW-SS-171 ACTGACGCTAGTGCATCACTTTCTGAGCCAGTGTTGCT		
JSW-SS-42 – 53 (with X8 barcode) CAAGCAGAAGACGGCATACGAGAT(X8)GTGACTGGAGTTCAGACGTGTGCTCTTCCGATCTACTGACGCTAGTGCATCA		
JSW-SS-54 – 61 (with X8 barcode) AATGATACGGCGACCACCGAGATCTACAC(X8)ACACTCTTTCCCTACACGACGCTCTTCCGATCT		
YL001 /5Phos/GATCGGAAGAG/3ddC/		
YL002-5 (with X4 barcode, Ns are mixed bases) AGCGGCAATTTCACACAGGACAAGCAGAAGACGGCATACGAGATNNNNNNNN(X4)GTGACTGGAGTTCAGACGTGTGCTCTTCCGATC*T		
YL006 AGCGGCAATTTCACACAGGA		
oAD493 ACACTGGCAGAGCATTACGCCCT		
YL009 CAAGCAGAAGACGGCATACGAG		
JBD-SS-273 – 280 (with X8 barcode, Ns are mixed bases) AATGATACGGCGACCACCGAGATCTACAC(X8)ACACTCTTTCCCTACACGACGCTCTTCCGATCTNNNNNNNNGCAGGGATGTCCACGAGGTCTCT		
**Other**		
Illumina MiSeq	Illumina	N/A
Illumina NextSeq	Illumina	N/A
Illumina NovaSeq	Illumina	N/A
