# Supplementary material for: Anti-restriction functions of injected phage proteins revealed by peeling back layers of bacterial immunity
Source: Nat Commun. 2025 Aug 22;16:7828. doi: 10.1038/s41467-025-63056-3 (PMC12373910; doi:10.1038/s41467-025-63056-3)
Supplement: Supplementary file 2 — Description of Additional Supplementary Files [file 41467_2025_63056_MOESM2_ESM.pdf]

Description of Additional Supplementary Files:

Supplementary Data 1: Experimental record of AG screens. A detailed record of all AG screens. The first sheet contains phage-host combinations, dates of the experiment, and preliminary results. The next three sheets each correspond to one replicate each of AG screening. Empirically measured multiplicity at time of infection, and dilutions used before plating are also recorded. Screens that had to be repeated for technical reasons are colorcoded.

Supplementary Data 2: Accessory regions of AGs selected for further study. Visualizations of ARs which contain AGs that produced counter-defense phenotypes in AG screens.

Supplementary Data 3: Experimental record of followup transposon whole-genome mutagenesis screens. The first sheet contains a detailed record of all follow-up transposon screens designed to phenocopy counterdefense phenotypes of AGs. These screens are performed with RB-TnSeq libraries prepared with naïve ECOR hosts (no AGs present). Each library is challenged by various phages one at a time. The timeline of each experiment indicates library growth characteristics. Empirically measured multiplicity at time of infection, and dilutions used before plating are also recorded. Screens were performed in two stages: first, a pilot with one phage for each RB-TnSeq library, and then a larger experiment with all other phages that showed enhanced infection upon expression of a counter-defense AG. The second sheet lists all hits recovered from the screens, including the ECOR17-Tn5 library challenged with various amounts of phage T4Δip2Δip3.

Supplementary Data 4: Host-AG combinations for affinity-purification/mass-spectrometry. Matrix of all host-AG combinations where potential binding partners of AGs were affinity-purified from the native host expressing counter-defense AGs, and identified by massspectrometry. Experiments were performed in triplicate. *Orf74* produced no phenotypes during AG screening and was used as a control for all experiments in the relevant hosts.

Supplementary Data 5: Pairwise alignments of GmrSD systems targeted by Ip1, Ip2, Ip3. Pairwise sequence alignments of GmrSDCT596 (Ip1 target) and GmrSDECOR21 (Ip2 target; BrxU), with GmrS1 and GmrSD2 components of GmrSDECOR17 (Ip3 target).

Supplementary Data 6: Clustering and genomic loci analysis of GmrSD sub-types in Enterobacteria. First sheet contains accessions of GmrS-like proteins in Enterobacteria grouped into clusters and sorted by abundance (most common clusters first). Second sheet contains genomic loci for all proteins in the first sheet. Third sheet contains details of putative Type IV REs selected for experiments.
