# Supplementary material for: Anti-restriction functions of injected phage proteins revealed by peeling back layers of bacterial immunity
Source: Nat Commun. 2025 Aug 22;16:7828. doi: 10.1038/s41467-025-63056-3 (PMC12373910; doi:10.1038/s41467-025-63056-3)
Supplement: Supplementary file 4 — Supplementary Dataset 2 [file 41467_2025_63056_MOESM4_ESM.pptx]

## Slide 1
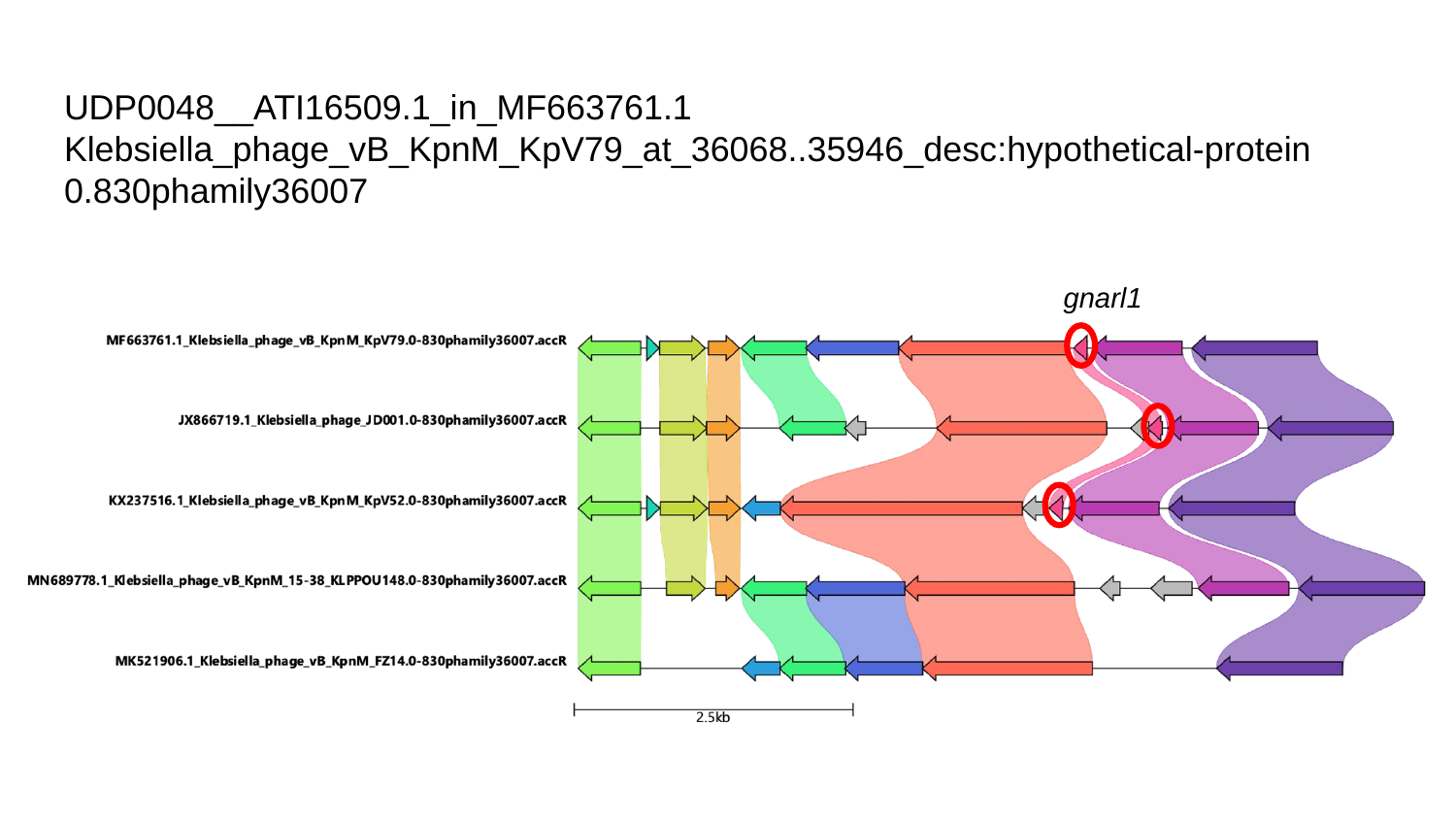

# UDP0048__ATI16509.1_in_MF663761.1
Klebsiella_phage_vB_KpnM_KpV79_at_36068..35946_desc:hypothetical-protein
0.830phamily36007
gnarl1

## Slide 2
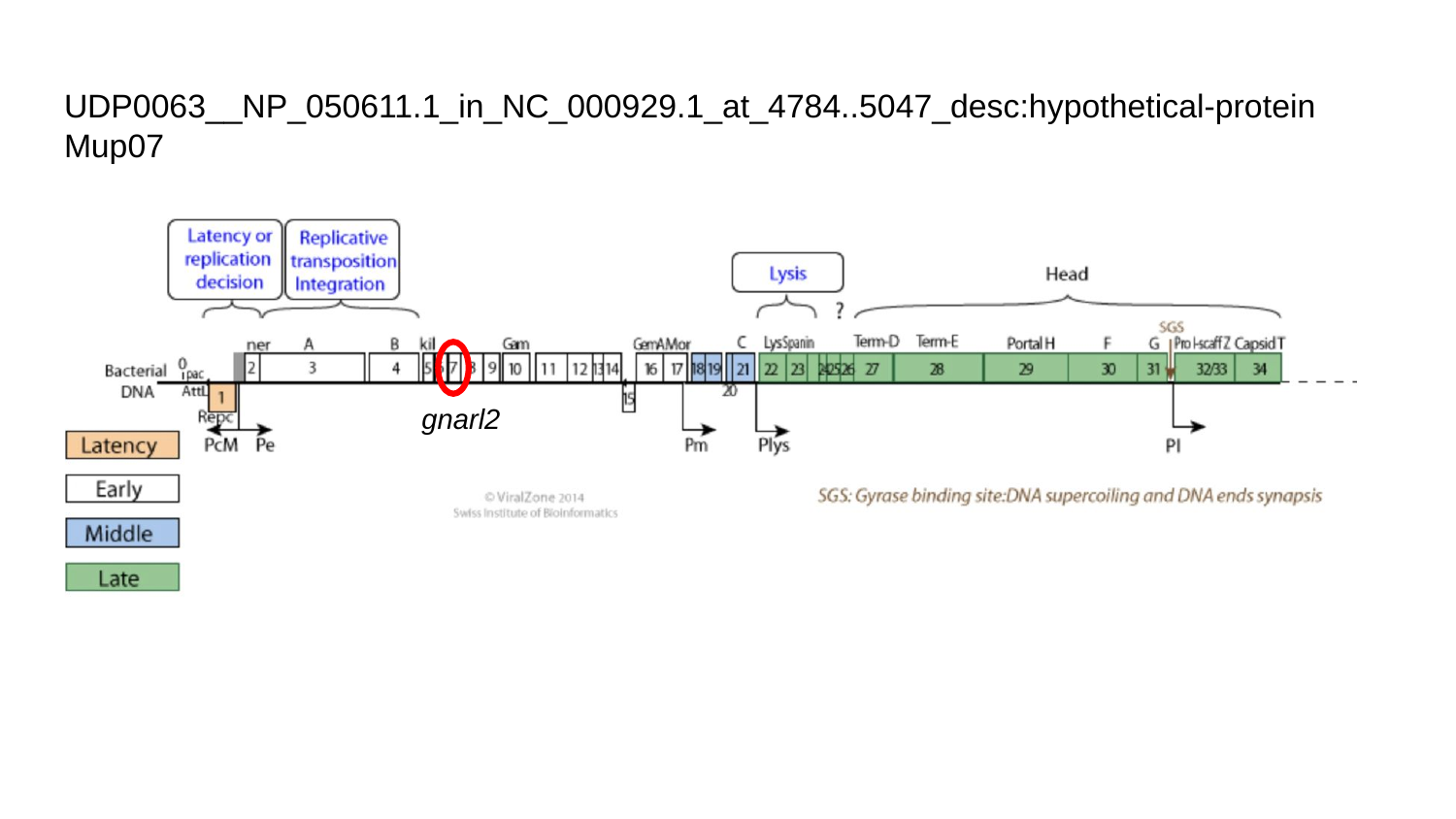

# UDP0063__NP_050611.1_in_NC_000929.1_at_4784..5047_desc:hypothetical-protein
Mup07
gnarl2

## Slide 3
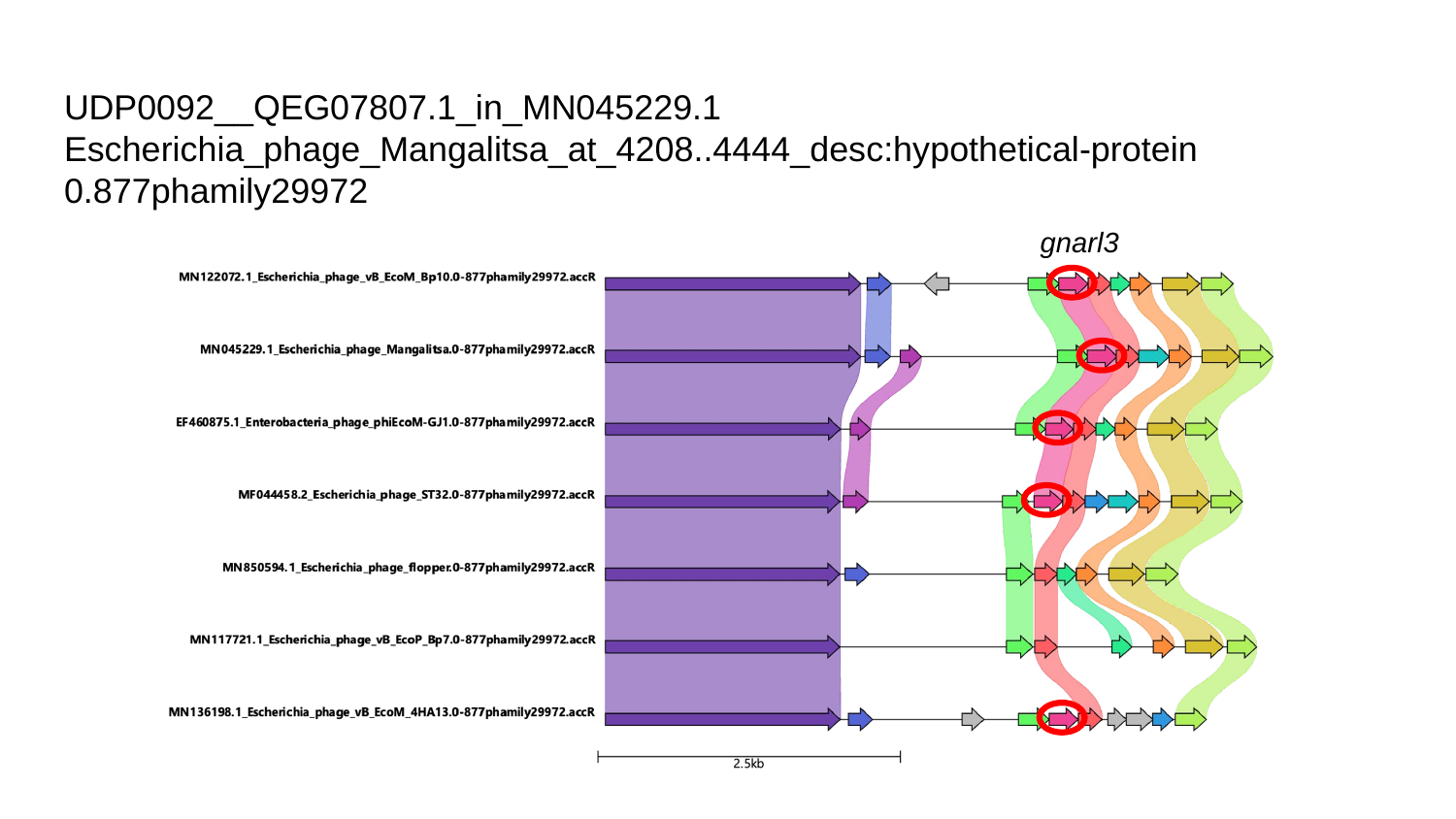

# UDP0092__QEG07807.1_in_MN045229.1
Escherichia_phage_Mangalitsa_at_4208..4444_desc:hypothetical-protein
0.877phamily29972
gnarl3

## Slide 4
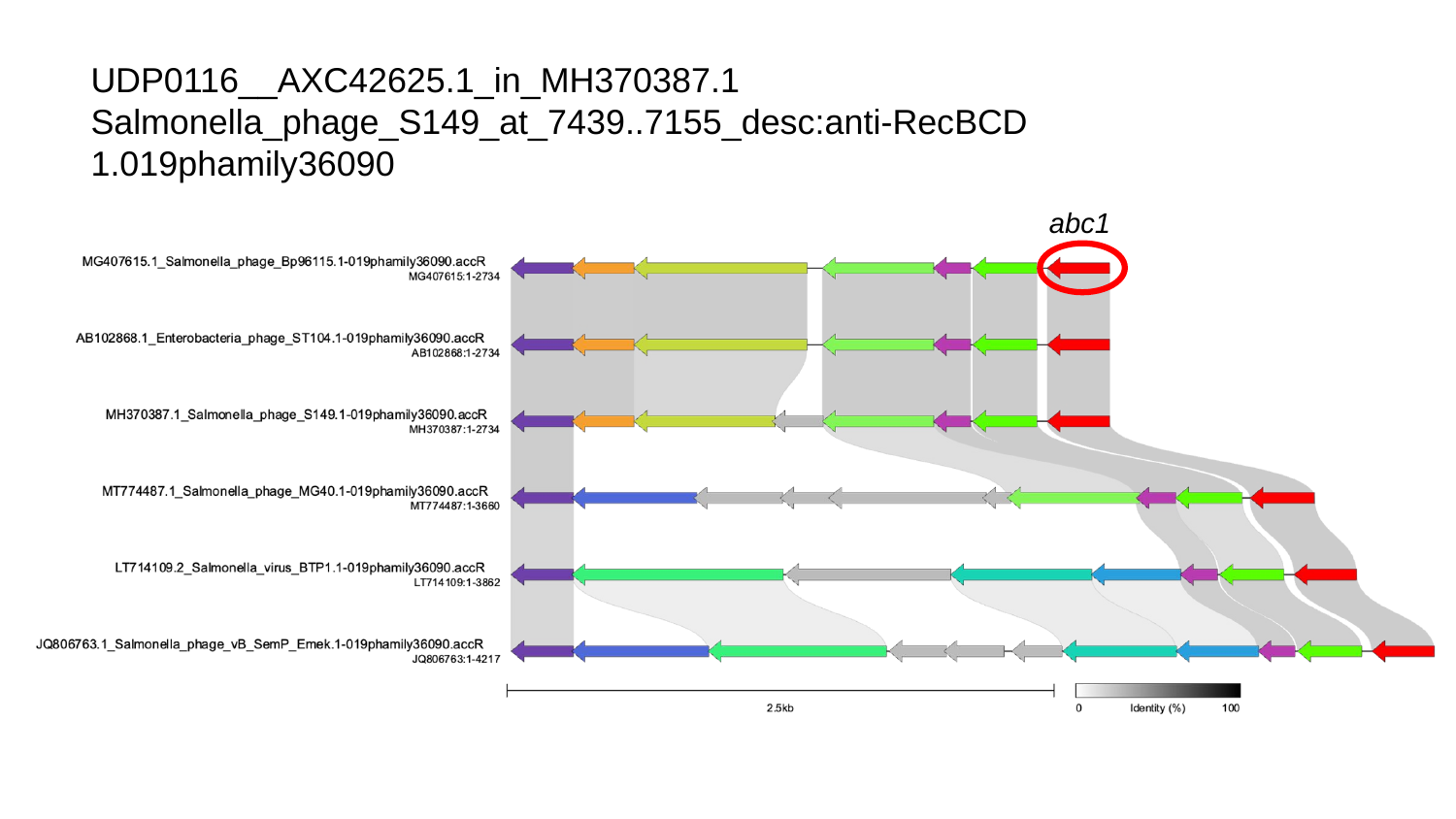

UDP0116__AXC42625.1_in_MH370387.1
Salmonella_phage_S149_at_7439..7155_desc:anti-RecBCD
1.019phamily36090
abc1

## Slide 5
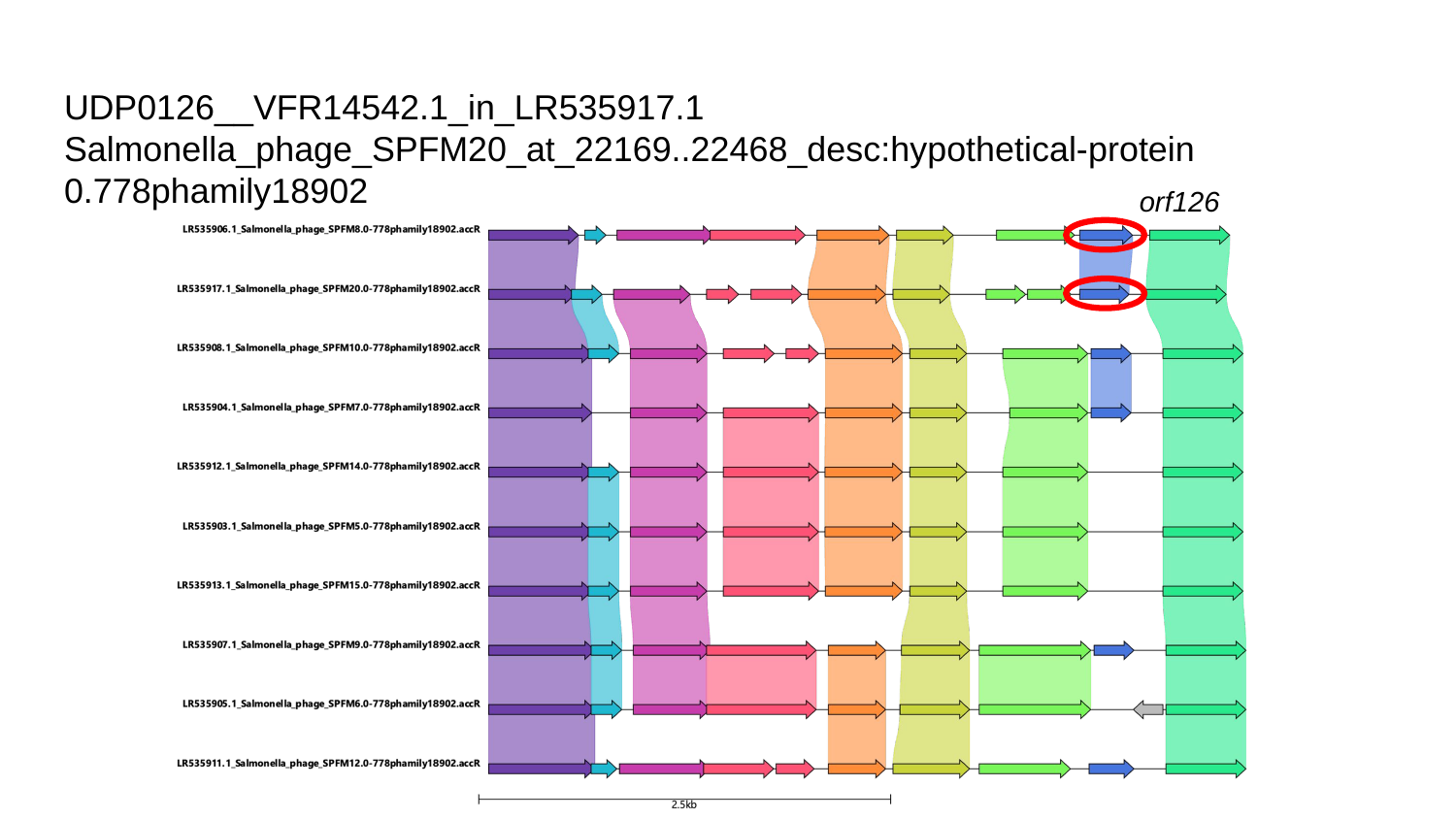

# UDP0126__VFR14542.1_in_LR535917.1
Salmonella_phage_SPFM20_at_22169..22468_desc:hypothetical-protein
0.778phamily18902
orf126

## Slide 6
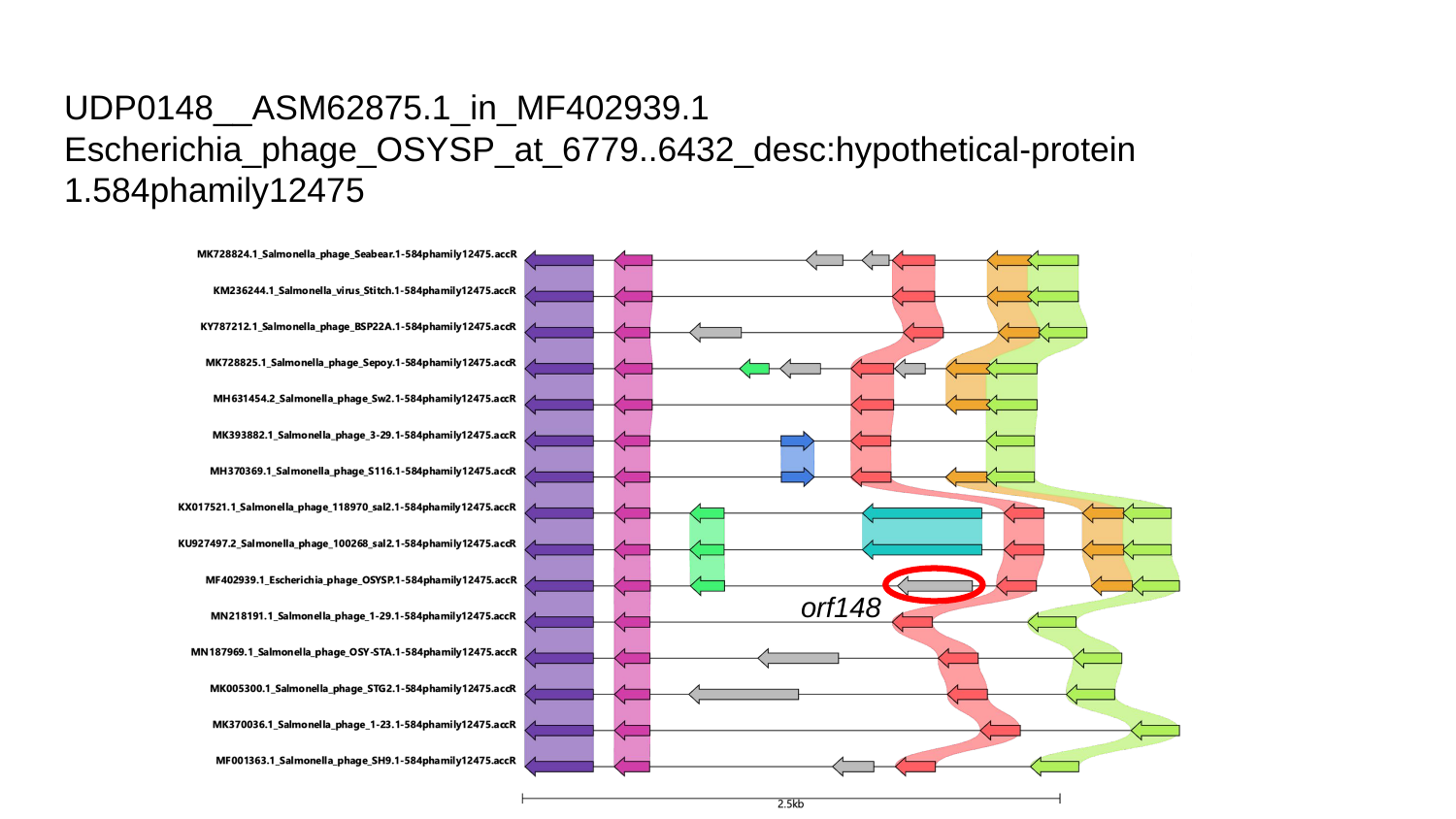

# UDP0148__ASM62875.1_in_MF402939.1
Escherichia_phage_OSYSP_at_6779..6432_desc:hypothetical-protein
1.584phamily12475
orf148
