# Supplementary figures and images for: Anti-restriction functions of injected phage proteins revealed by peeling back layers of bacterial immunity
Source: Nat Commun. 2025 Aug 22;16:7828. doi: 10.1038/s41467-025-63056-3 (PMC12373910; doi:10.1038/s41467-025-63056-3)

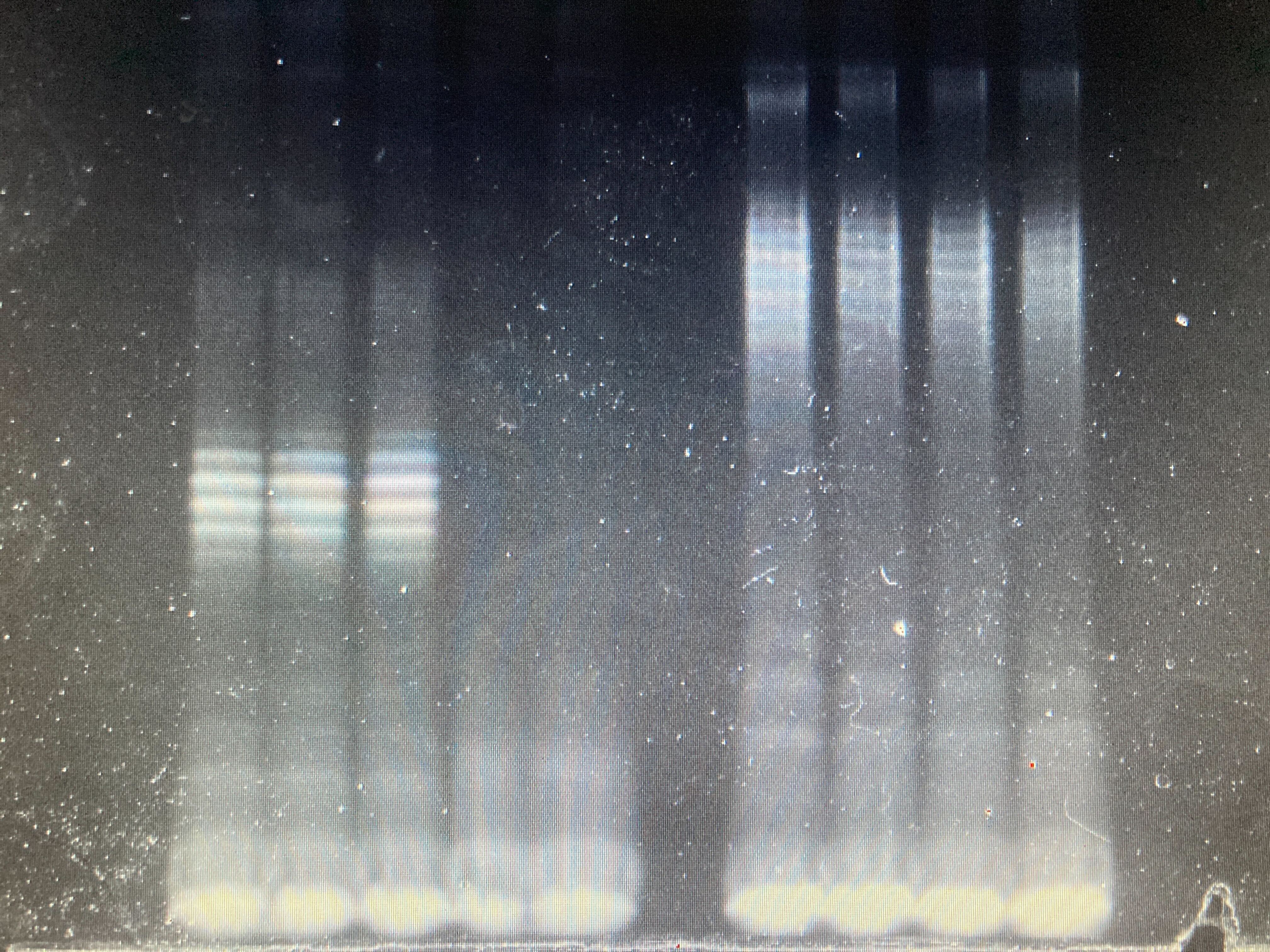

Supplement: Supplementary file 11 — Source data [file 41467_2025_63056_MOESM11_ESM.zip › Source Data/3C LPS gel.jpg]

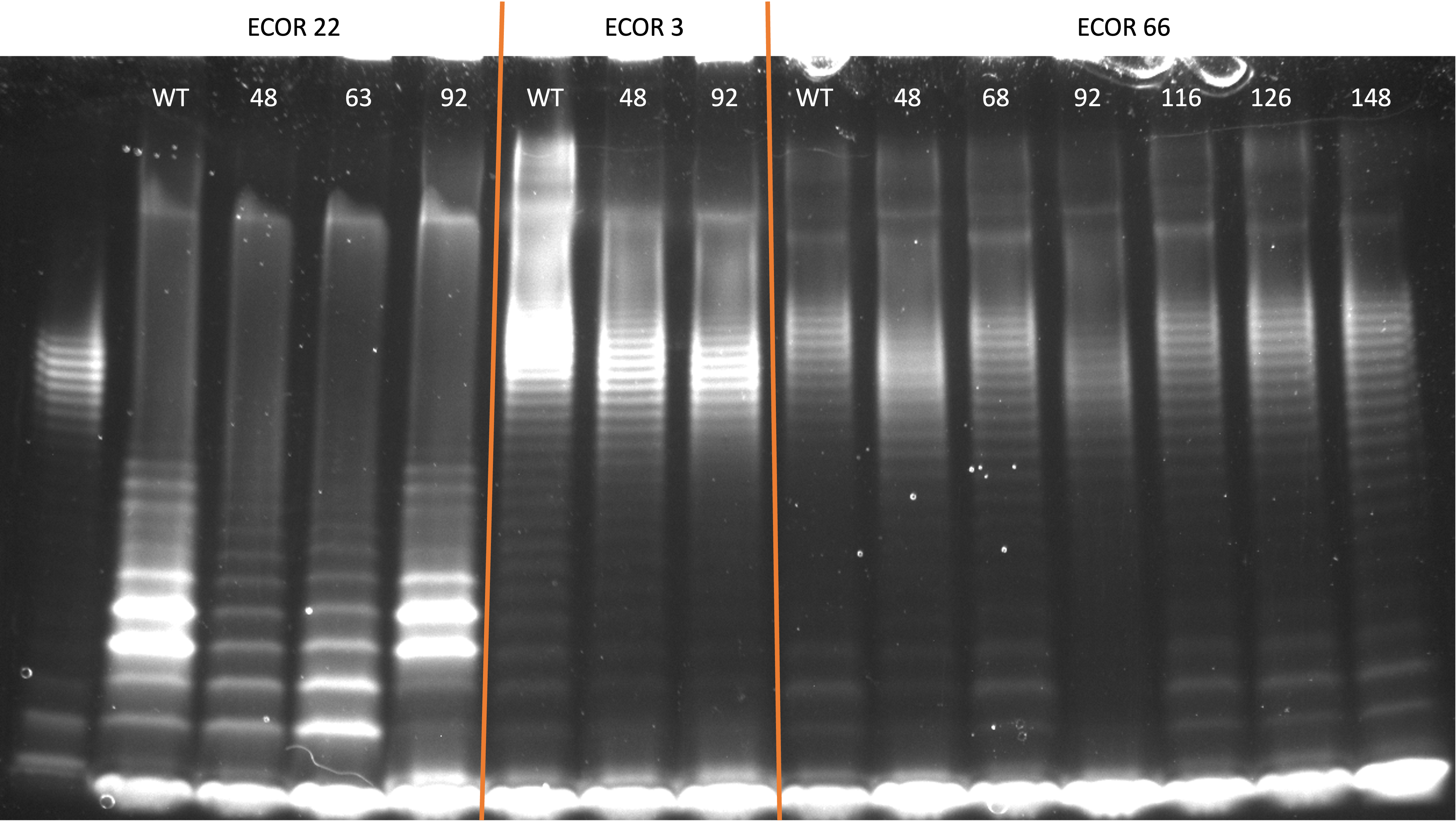

Supplement: Supplementary file 11 — Source data [file 41467_2025_63056_MOESM11_ESM.zip › Source Data/2C-2 LPS gel.png]

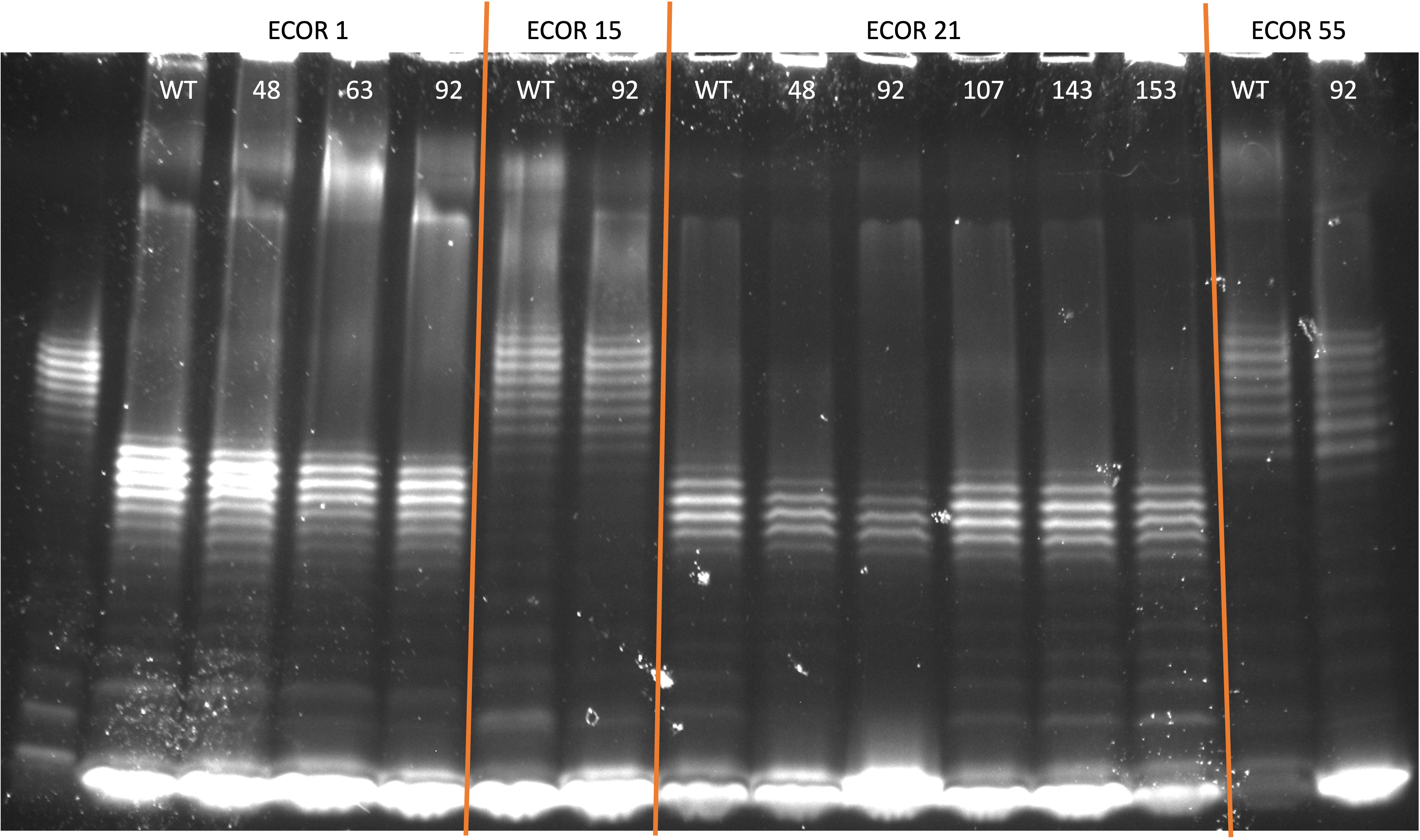

Supplement: Supplementary file 11 — Source data [file 41467_2025_63056_MOESM11_ESM.zip › Source Data/2C-1 LPS gel.png]

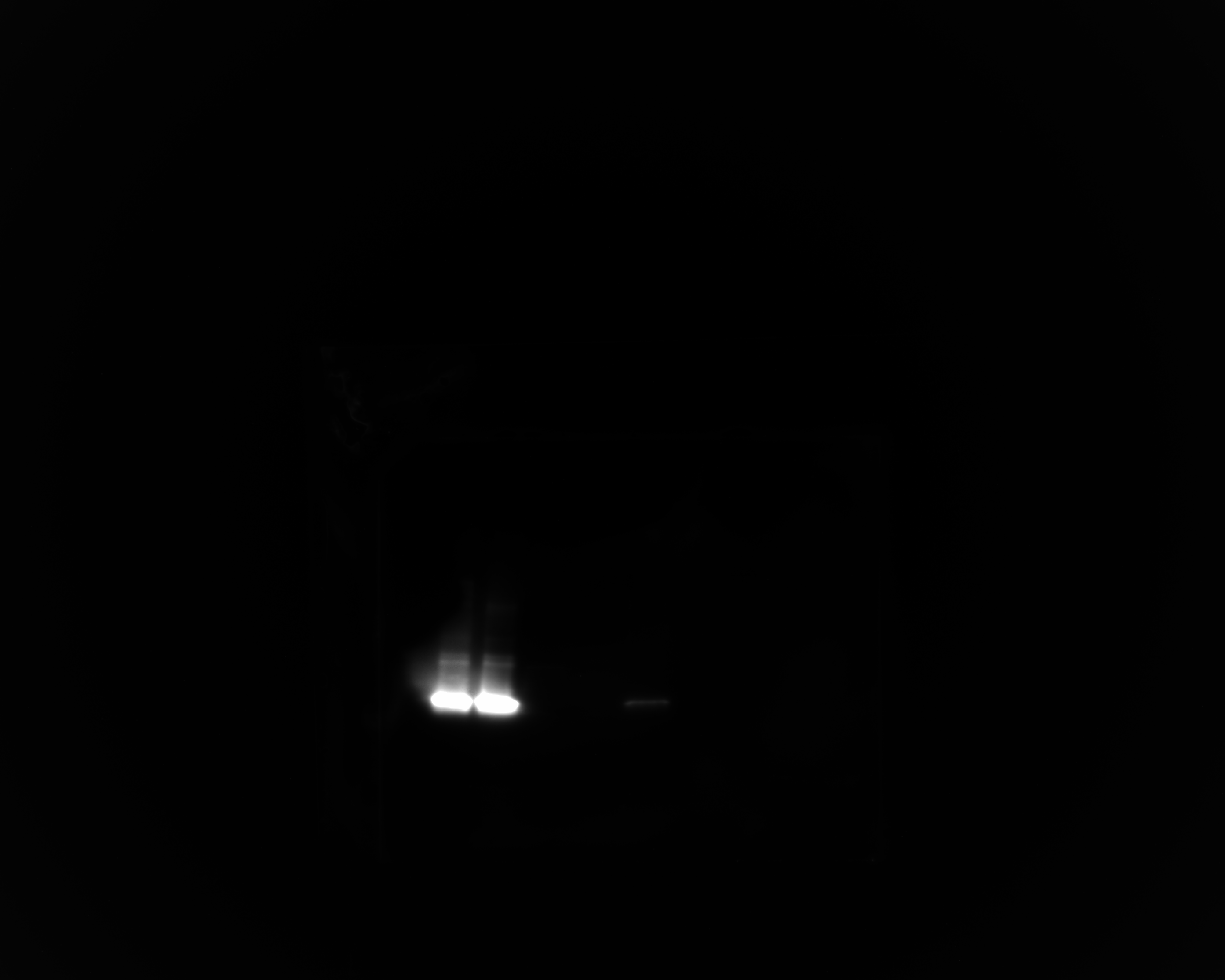

Supplement: Supplementary file 11 — Source data [file 41467_2025_63056_MOESM11_ESM.zip › Source Data/2E anti-FLAG 1.tif]

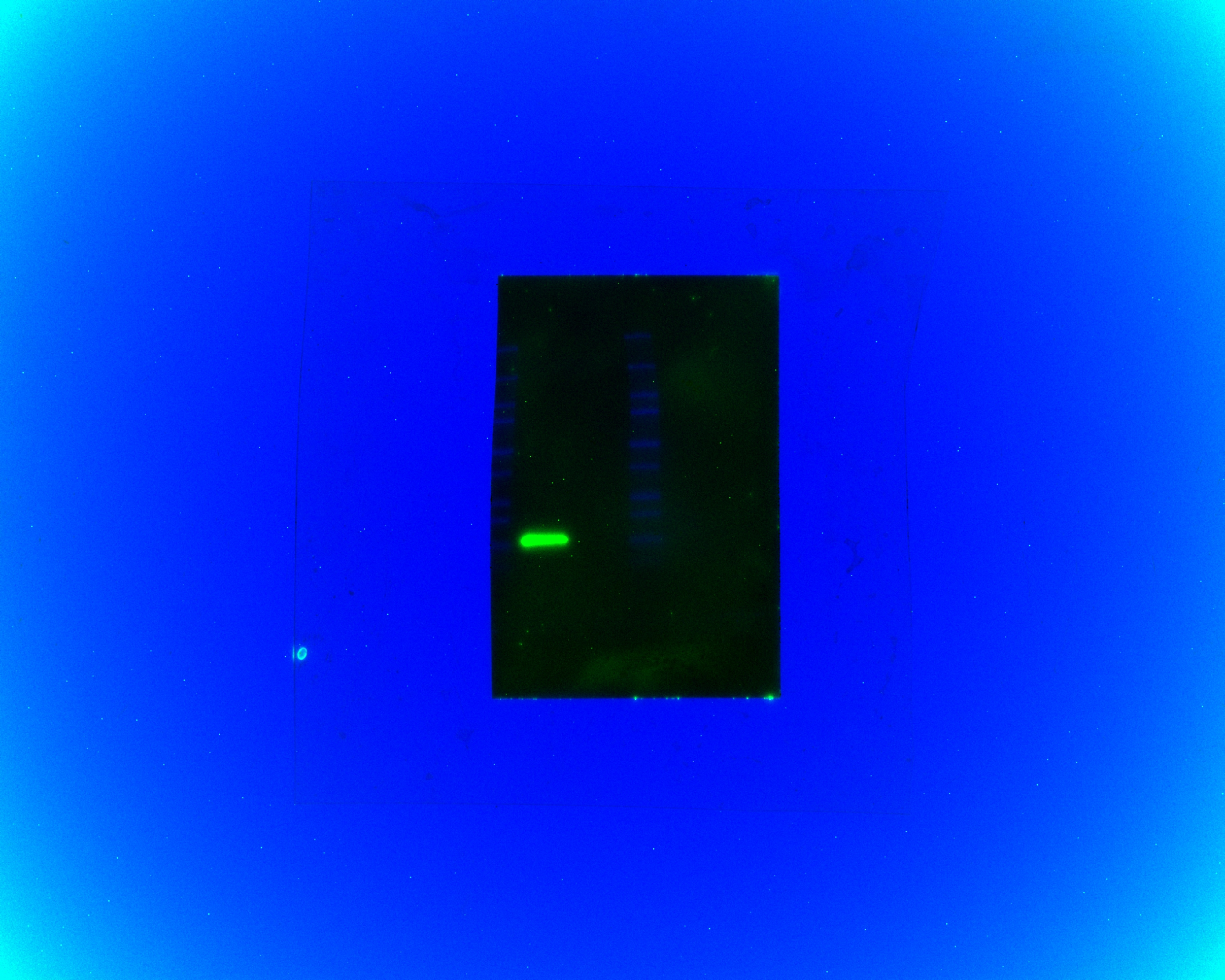

Supplement: Supplementary file 11 — Source data [file 41467_2025_63056_MOESM11_ESM.zip › Source Data/2E anti-FLAG composite.jpg]

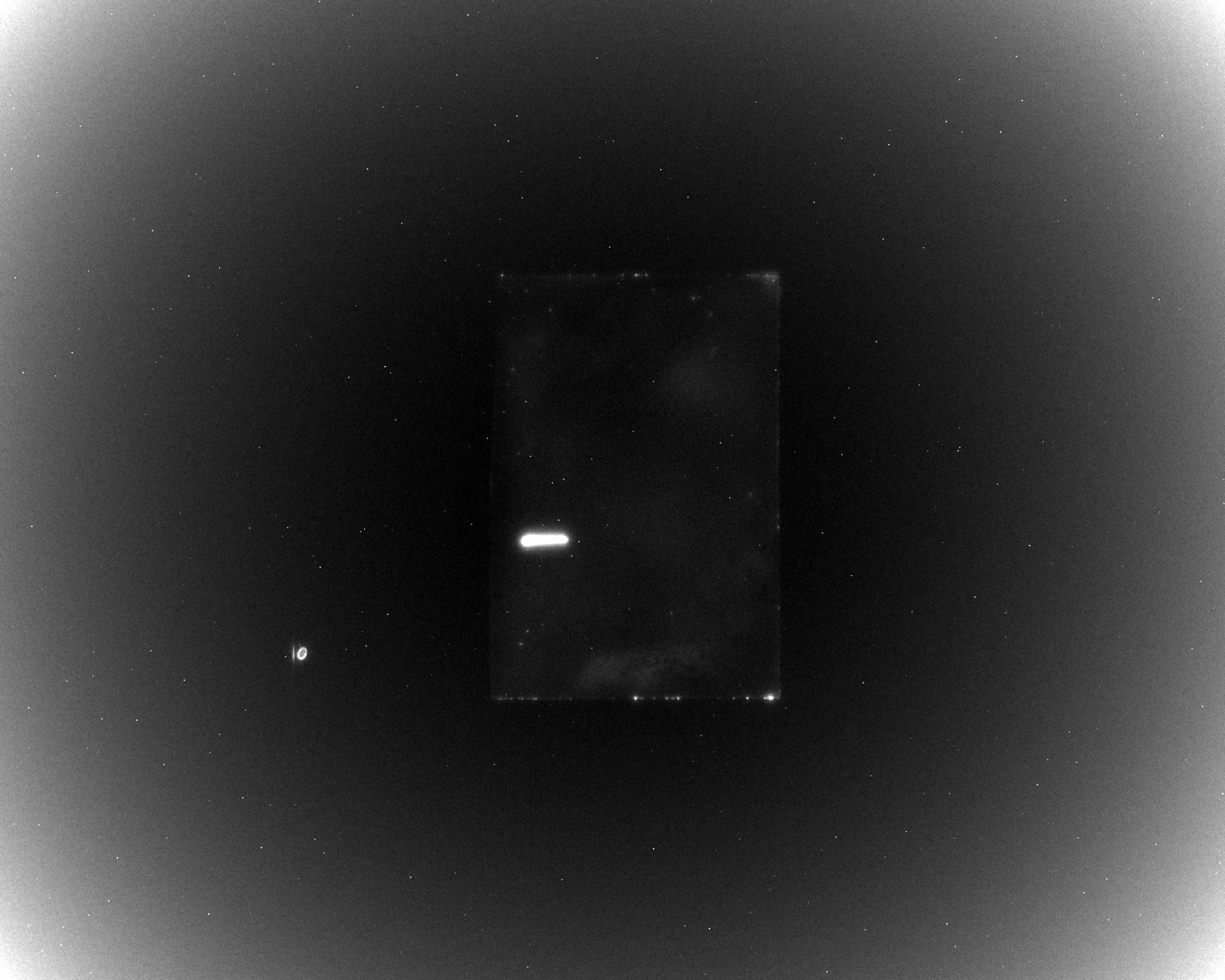

Supplement: Supplementary file 11 — Source data [file 41467_2025_63056_MOESM11_ESM.zip › Source Data/2E anti-FLAG 2.tif]

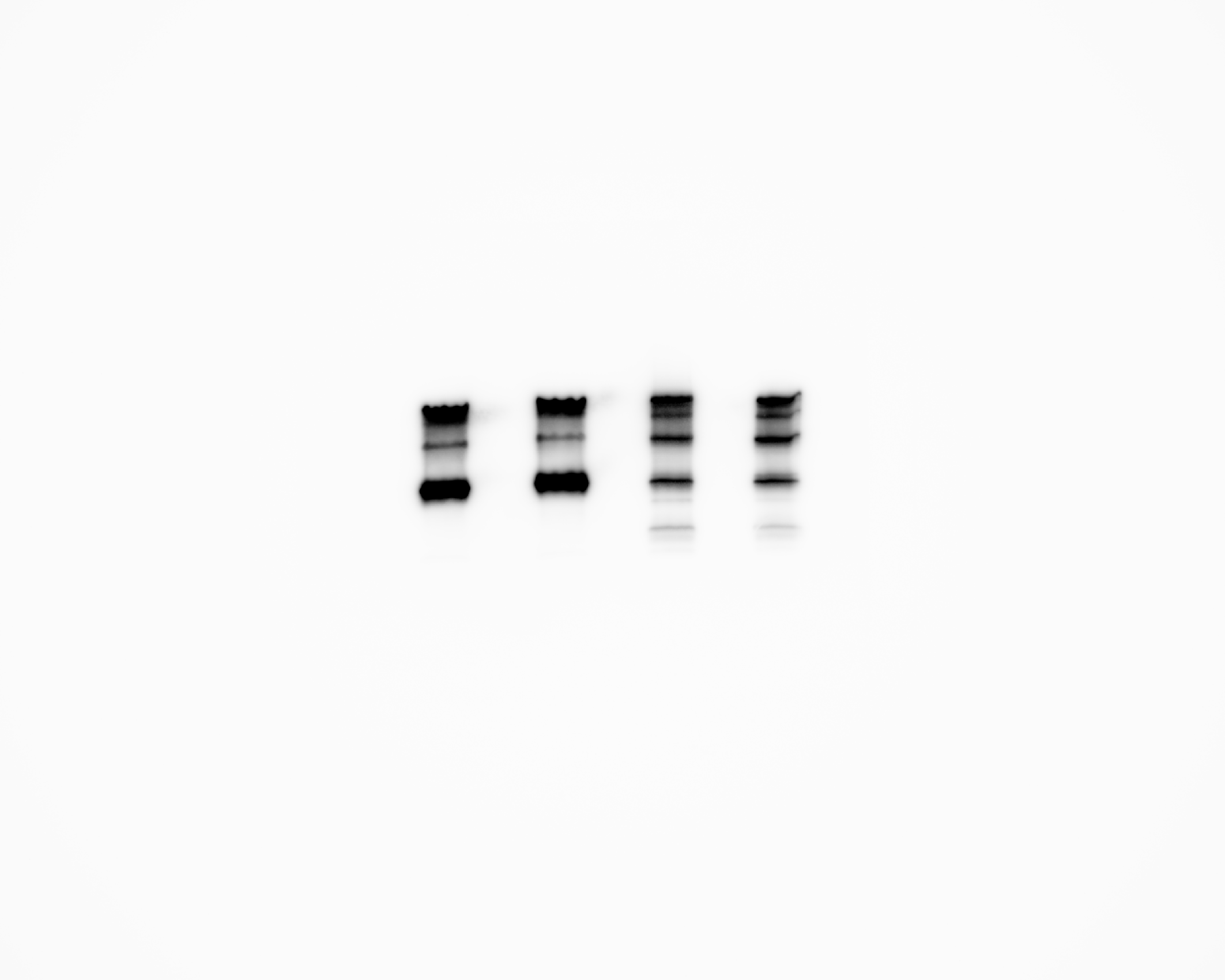

Supplement: Supplementary file 11 — Source data [file 41467_2025_63056_MOESM11_ESM.zip › Source Data/2E anti-GST.tif]

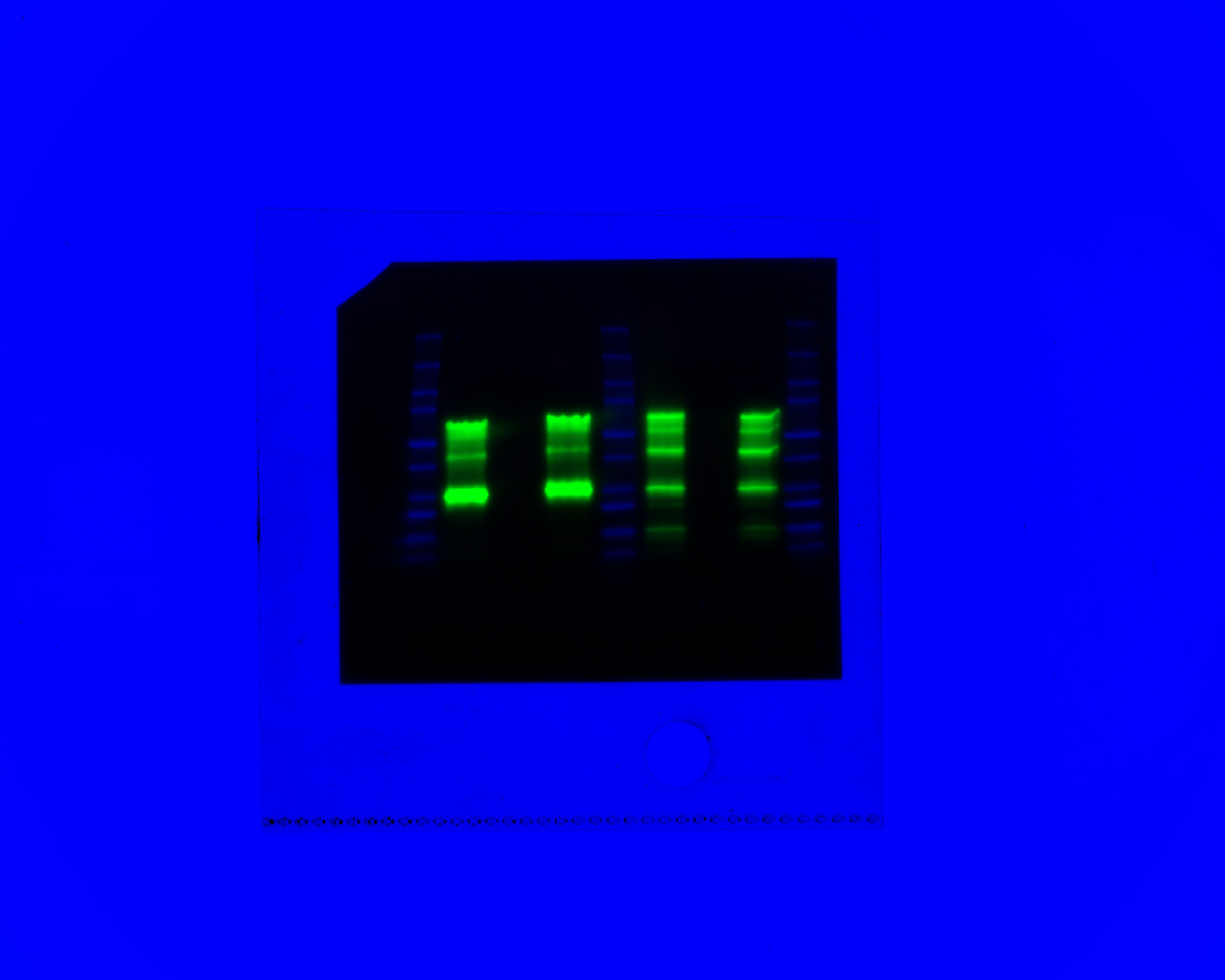

Supplement: Supplementary file 11 — Source data [file 41467_2025_63056_MOESM11_ESM.zip › Source Data/2E anti-GST composite.tif]
